# Disruption of exon-bridging interactions between the minor and major spliceosomes results in alternative splicing around minor introns

**DOI:** 10.1093/nar/gkab118

**Published:** 2021-02-28

**Authors:** Anouk M Olthof, Alisa K White, Stephen Mieruszynski, Karen Doggett, Madisen F Lee, Almahdi Chakroun, Alice K Abdel Aleem, Justine Rousseau, Cinzia Magnani, Chaim M Roifman, Philippe M Campeau, Joan K Heath, Rahul N Kanadia

**Affiliations:** Physiology and Neurobiology Department, University of Connecticut, 75 N. Eagleville Road, Storrs, CT 06269, USA; Physiology and Neurobiology Department, University of Connecticut, 75 N. Eagleville Road, Storrs, CT 06269, USA; Epigenetics and Development Division, Walter and Eliza Hall Institute of Medical Research, Parkville, VIC 3052, Australia; Epigenetics and Development Division, Walter and Eliza Hall Institute of Medical Research, Parkville, VIC 3052, Australia; Physiology and Neurobiology Department, University of Connecticut, 75 N. Eagleville Road, Storrs, CT 06269, USA; Neurogenetics, Weill Cornell Medicine—Qatar, Doha, Qatar; Neurogenetics, Weill Cornell Medicine—Qatar, Doha, Qatar; CHU Sainte-Justine Research Center, Montreal, QC H3T 1C5, Canada; Neonatology and Neonatal Intensive Care Unit, Maternal and Child Department, University of Parma, Parma, 43121, Italy; Division of Immunology and Allergy, Department of Pediatrics, The Hospital for Sick Children and the University of Toronto, Toronto, ON M5G 1X8, Canada; The Canadian Centre for Primary Immunodeficiency and The Jeffrey Modell Research Laboratory for the Diagnosis of Primary Immunodeficiency, The Hospital for Sick Children, Toronto, ON M5G 1X8, Canada; Department of Pediatrics, University of Montreal, Montreal, QC H4A 3J1, Canada; Epigenetics and Development Division, Walter and Eliza Hall Institute of Medical Research, Parkville, VIC 3052, Australia; Physiology and Neurobiology Department, University of Connecticut, 75 N. Eagleville Road, Storrs, CT 06269, USA; Institute for System Genomics, University of Connecticut, Storrs, CT 06269, USA

## Abstract

Vertebrate genomes contain major (>99.5%) and minor (<0.5%) introns that are spliced by the major and minor spliceosomes, respectively. Major intron splicing follows the exon-definition model, whereby major spliceosome components first assemble across exons. However, since most genes with minor introns predominately consist of major introns, formation of exon-definition complexes in these genes would require interaction between the major and minor spliceosomes. Here, we report that minor spliceosome protein U11-59K binds to the major spliceosome U2AF complex, thereby supporting a model in which the minor spliceosome interacts with the major spliceosome across an exon to regulate the splicing of minor introns. Inhibition of minor spliceosome snRNAs and U11-59K disrupted exon-bridging interactions, leading to exon skipping by the major spliceosome. The resulting aberrant isoforms contained a premature stop codon, yet were not subjected to nonsense-mediated decay, but rather bound to polysomes. Importantly, we detected elevated levels of these alternatively spliced transcripts in individuals with minor spliceosome-related diseases such as Roifman syndrome, Lowry–Wood syndrome and early-onset cerebellar ataxia. In all, we report that the minor spliceosome informs splicing by the major spliceosome through exon-definition interactions and show that minor spliceosome inhibition results in aberrant alternative splicing in disease.

## INTRODUCTION

The splicing of introns by the spliceosome, a ribonucleoprotein (RNP) complex, is a crucial step in the regulation of eukaryotic gene expression. Most eukaryotes contain two types of spliceosomes that each recognize their own class of introns. The canonical spliceosome, also called the major spliceosome, splices U2-type or major introns, and consists of the small nuclear RNPs (snRNPs) U1, U2, U4, U6 and U5 (1–3). Conversely, a small subset of introns called U12-type or minor introns contain divergent consensus sequences and are spliced by the minor spliceosome, consisting of the snRNPs U11, U12, U4atac, U6atac and U5 (4,5). Even though four of the five snRNAs differ between the two spliceosomes, the splicing reaction executed by each spliceosome is analogous. For both intron classes, the first step consists of recognition of the 5′ splice site (SS) and branch point sequence (BPS) by either base pairing of the U1 and U2 snRNPs of the major spliceosome or U11/U12 di-snRNP of the minor spliceosome (6). The recognition of the correct BPS in vertebrates is complicated by the fact that introns are several kilobases long (7). Consequently, a single intron can possess many putative BPSs. As such, identification of the 3′ end of most introns in vertebrates is thought to occur through exon-spanning interactions (8,9). For major introns, formation of the exon bridge starts with recognition and binding of the 5′SS by U1 snRNP. This then facilitates the recruitment of the U2AF complex to the polypyrimidine tract of the upstream intron, and binding of U2 snRNP to the correct BPS (10,11). Afterward, the tri-snRNP U4/U6.U5 is recruited, which is followed by several remodeling steps that result in two sequential transesterification reactions (12,13).

While the exon-definition model elegantly explains the splicing of major introns, it is unclear how this model extends to minor introns, which are often flanked by major introns (14). Inherent to this unique gene organization, the exon-definition model would posit that the minor and major spliceosomes must interact with each other to successfully splice the minor intron and flanking major introns. Specifically, after U11 snRNP binds to the 5′SS of a minor intron, it would be predicted to bind to the U2AF complex to recruit U2 snRNP to the BPS of the upstream major intron. Similarly, binding of U1 snRNP to a downstream major 5′SS would be predicted to enhance base pairing of U12 snRNA with the minor-type BPS, followed by recruitment of the U4atac/U6atac.U5 tri-snRNP and splicing of the minor intron. Indeed, it has been shown that binding of U1 snRNP to a downstream major 5′SS can enhance the splicing efficiency of a minor intron (15). Moreover, several reports have suggested that major and minor spliceosome components can interact with each other, although it remains unknown how U11 snRNP would engage with U2 snRNP (16,17).

Here, we wanted to test whether the exon-definition model would extend to the splicing of 2% of the genes in the human genome that contain minor introns. To this end, we evaluated the effect of minor spliceosome snRNA inhibition on the splicing of minor introns in cell culture and *in vivo*, by employing antisense morpholinos and our U11 conditional knockout mouse. Here, we report that inhibition of all minor spliceosome snRNAs results in elevated alternative splicing (AS) around minor introns, particularly skipping of the exons flanking the minor intron. Since exon skipping is the predicted outcome of disrupted exon-definition interactions between the major and minor spliceosomes, we next sought to understand how the spliceosomal complexes interacted. We found that PDCD7 (U11-59K), a protein component of the U11 snRNP, directly interacts with the U2AF complex and other protein components of the U2 snRNP, suggesting that it is involved in the exon bridge between major and minor introns. Importantly, we found that aberrant minor intron-containing gene (MIG) transcripts that are produced upon disruption of exon-definition interactions are bound to polysomes and not subjected to nonsense-mediated decay (NMD), suggesting they are translated. Finally, mechanistic insight into the splicing of minor introns can also aid in our understanding of diseases that are linked to mutations in minor spliceosome components. Indeed, we detected alternatively spliced MIG transcripts in peripheral blood mononuclear cells (PBMCs) from individuals with Roifman syndrome (*RNU4ATAC*), Lowry–Wood syndrome (*RNU4ATAC*) and early-onset cerebellar ataxia (*RNU12*), suggesting they contribute to disease pathogenesis. In all, our findings provide insight into the exon-definition interactions that normally take place to regulate the proper splicing of introns in MIGs and show the consequences of disrupting these interactions in disease.

## MATERIALS AND METHODS

### Animal husbandry

All mouse procedures were performed according to the protocols approved by the University of Connecticut Institutional Animal Care and Use Committee, which ensures adherence to the U.S. Public Health Service Policy on the treatment of laboratory animals. The generation of the U11 cKO mouse has been described previously (18). To ablate U11 snRNA in the developing cortex, we crossed the *Rnu11* cKO mouse to *Emx1-Cre^+^* mice (19). These crosses resulted in control (*Rnu11*^WT/Flx^::*Emx1*-Cre^+/−^) and U11 cKO embryos (*Rnu11*^Flx/Flx^::*Emx1*-Cre^+/−^). To isolate RNA bound to polysomes, we crossed our U11 cKO mice with *Rpl22*-HA^+^ mice and harvested E14 embryos (20).

### Human subjects

Informed consent was obtained from individuals with mutations in *RNU4ATAC* (*N* = 2), their unaffected carrier parents (*N =* 3) and unrelated healthy controls (*N* = 3) using a protocol approved by the Institutional Review Board committee at the CHU Sainte-Justine. The unaffected carriers were the fathers of the patients and possessed one mutant *RNU4ATAC* allele, but were asymptomatic. All individuals with Roifman syndrome and/or Lowry–Wood syndrome were autosomal recessive for *RNU4ATAC*, but contained two different mutations, which is referred to as a compound heterozygote. The phenotypic description of the individual with Lowry–Wood syndrome described in this manuscript had previously been published (21,22). In contrast, one of the individuals with Roifman syndrome described in this manuscript had not previously been reported. This individual experienced asymmetric intra-uterine growth retardation during pregnancy along with other ultrasound anomalies (including a suspected aortic coarctation, which was later not confirmed). Prenatal investigations revealed a normal array comparative genome hybridization and a customized Noonan syndrome panel revealed an inherited *KMT2D* variant of uncertain significance. Given that this variant was inherited, it was deemed non-pathogenic. The patient was born at term with a weight (2320 g) and length (42 cm) both below the third percentile. Moreover, the head circumference was at the 50th percentile. He had micromelia and brachydactyly, and a skeletal survey showed platyspondyly, irregular metaphyses and delayed epiphyseal ossification [i.e. a clinical presentation of spondyloepimetaphyseal dysplasia (SEMD)]. A SEMD panel then revealed two *RNU4ATAC* mutations *in trans* (Supplementary Figure S7A), which have previously been reported in another individual with Roifman syndrome (23). The patient was then evaluated by additional clinicians who determined the presence of low titers of antibodies against tetanus following immunization. Therefore, he is now, at 1.5 years of age, supplemented with immunoglobins. He has not had a serious infection to date and is doing well.

The individuals with mutation in *RNU12* (*N* = 5) and their unaffected carrier parents (*N* = 3) mentioned in this manuscript have been described previously (24). Informed consent from these families was obtained using protocols approved by Institutional Review Board committee at Weill Cornell Medicine—Qatar and Hamad Medical Corporation.

### Cell culture

HEK293T cells were cultured in Dulbecco’s modified Eagle’s medium (DMEM), supplemented with 10% fetal bovine serum (FBS), 5% sodium pyruvate and penicillin (100 IU/ml) and streptomycin (100 μg/ml). Cells were maintained at 37°C and 5% CO_2_.

Human lung adenocarcinoma A549 cells were cultured in RPMI 1640 medium supplemented with 10% FBS, 2 mM l-glutamine and penicillin (100 IU/ml) and streptomycin (100 μg/ml), and grown at 37°C in a 10% CO_2_ humidified atmosphere. Cell lines were authenticated using small tandem repeat profiling (Cell Bank Australia, Report #19-338).

### Telencephalon culture

Telencephalons of E12 control and U11 cKO embryos were dissected in ice-cold 1× HBSS. They were then individually placed on 22-μm micropore filters in a six-well plate filled with culture medium (1× DMEM/F-12, 1× penicillin/streptomycin, 1× G5 supplement, 2× B27 supplement). The culture medium of one telencephalon of each embryo was then supplemented with 30 μg/ml cycloheximide. Finally, the tissues were cultured for 2 h at 37°C and 5% CO_2_ and collected in 100 μl TRIzol.

### siRNA transfections

To knock down splicing factors in HEK293T cells, we obtained a customized library containing ON-TARGET plus SMARTpool siRNAs from Dharmacon Inc. siRNAs were resuspended in UP H_2_O to a concentration of 2 μM. Fifty picomoles of siRNA against each gene combined with 500 ng pCAG-Mlst8 splicing reporter was co-transfected in 24-well plates using DharmaFECT DUO, as per the manufacturer’s guidelines (Dharmacon Inc., #T-2010-01). After 48 h, cells were harvested in 100 μl TRIzol or 100 μl modified RIPA buffer (50 mM Tris–HCl, pH 8.0, 150 mM NaCl, 0.5% SDS and 1× protease inhibitor). To repeat the experiments with *N*-value, we obtained siGenome SMARTpool siRNAs for *PDCD7* (#M-012096-00-0005), *RNPC3* (#M-021646-01-0005) and *ZRSR2* (#M-006596-01-0005) and followed the same experimental paradigm as described before.

To knock down PDCD7, A549 cells were transfected in a six-well plate with either 50 nM of two independent human PDCD7 ON-TARGETplus siRNAs (Dharmacon Inc., #J-012096-05-0010 and J-012096-06-0010) or a non-targeting control siRNA (Dharmacon Inc., #D-001810-01-05) using DharmaFECT 1 (Dharmacon Inc., #T-2001-03) according to the manufacturer’s protocol alongside a non-transfected control. siRNA transfections were carried out in triplicate and treated as independent replicates hereafter. Seventy-two hours after transfection, cells were harvested with 10% of the sample processed for RNA and 90% for nuclear protein extraction.

### Plasmids and splicing constructs

The pCAG-GFP plasmid (Addgene, #11150) was obtained from Addgene and utilized for the design of the pCAG-Mlst8 splicing reporter. First, the pCAG-GFP plasmid was cut with EcoRI and NotI restriction enzymes to remove the GFP cassette. The four exons and three introns of Mlst8 were PCR amplified with primers listed in Supplementary Table S1 and then cloned into the pCAG backbone using the Gibson Assembly Master Mix (NEB, #E2611S). The pCMV6-Pdcd7-Myc plasmid was obtained from OriGene (#MR214517).

### Morpholino electroporation

Electroporations were performed as described previously (25). Briefly, control (5′-CCTCTTACCTCAGTTACAATTTATA-3′) (26), U12 (5′-TCGTTATTTTCCTTACTCATAAGTT-3′) (25), U4atac (5′-CAGGCGTTAGCAGTACTGCCCTCAC-3′), U6atac (5′-AACCTTCTCTCCTTTCATACAACAC-3′) (26) and U2 (5′-TGATAAGAACAGATACTACACTTGA-3′) (25) antisense morpholino oligonucleotides (MOs) were obtained from GeneTools, Inc. and dissolved in water to a final concentration of 0.5 nmol/μl. Approximately 1–2 × 10^4^ cells were used for each separate electroporation (*N =* 3 per electroporation condition). Cells were washed with phosphate-buffered saline (PBS), followed by mixing with 15 nmol of morpholino in a 1.5-ml Eppendorf tube. After a 10 min incubation at room temperature, cells were transferred to a 4-mm-gap electroporation cuvette and electroporated using a Bio-Rad Gene Pulser at 200 V for 50 ms. Cells were then plated in 10 ml growth medium for 6 h. Afterward, cells were washed once in PBS and plated in new growth medium supplemented with 200 μM 5′-ethynyl uridine (EU) (Thermo Fisher Scientific, #C10365). Two hours later, cells were harvested and homogenized in 1 ml TRIzol (Thermo Fisher Scientific, #15596018), followed by extraction of total RNA. For isolation of nascently transcribed, EU-pulsed RNA, 5 μg of total RNA was biotinylated using the Click-IT kit (Thermo Fisher Scientific, #C10365), as per the manufacturer’s instructions. Then, 50 ng of biotinylated RNA was pulled down on Dynabeads (Thermo Fisher Scientific, #C10365) and resuspended in 5 μl buffer. The nascently transcribed RNA was then immediately used for library preparation.

### Immunoprecipitation

#### Polysomes

Ribosomal-bound RNA was extracted from the dorsal telencephalons of E14 *Rnu11*^WT/Flx^::*Emx1*-Cre^+/−^::*Rpl22*-HA^+/−^ and *Rnu11*^Flx/Flx^::*Emx1*-Cre^+/−^::*Rpl22*-HA^+/−^ embryos as described previously (20). Briefly, for each litter, the dorsal telencephalons of all embryos of each genotype (*N* = 4) were pooled and used for Dounce homogenization in 1 ml polysome buffer (50 mM Tris, pH 7.5, 100 mM KCl, 12 mM MgCl_2_, 1% Igepal, 1 mM DTT, 200 U/ml RNase inhibitor, 1 mg/ml heparin, 100 μg/ml cycloheximide, 1× protease inhibitor). Fifty microliters of the Dounce homogenized sample was labelled as ‘input’ and added to 350 μl RLT buffer with 1% 2-mercaptoethanol (Qiagen), followed by RNA extraction. The remaining sample was centrifuged and the supernatant was extracted. This was then added to HA-coupled protein G Dynabeads (Thermo Fisher Scientific, #10004D) and rotated overnight at 4°C. Afterward, beads were washed three times in high-salt buffer (50 mM Tris, pH 7.5, 300 mM KCl, 12 mM MgCl_2_, 1% Igepal, 1 mM DTT, 100 μg/ml cycloheximide) and 350 μl RLT buffer with 1% 2-mercaptoethanol was added to the beads. After vigorous vortexing, the beads were immobilized and supernatant was labeled as ‘IP’. Figure 4C contains representative images of results that have been repeated at least four times.

#### Pdcd7-Myc

To identify which proteins Pdcd7 interacts with, we overexpressed a pCMV6-Pdcd7-Myc plasmid (OriGene, #MR214517) in HEK293T cells using GenJet II (SignaGen, #SL100489), as per the manufacturer’s guidelines. As a control, an empty vector was utilized. After 48 h, cells were washed three times in 1× PBS and cross-linked with UV (365 nm) using the Stratalinker 2400. Settings were set to 4000 J/cm^2^, followed by 2000 J/cm^2^. Cells were dislodged and resuspended in 400 μl modified RIPA buffer (50 mM Tris–HCl, pH 8.0, 150 mM NaCl, 0.5% SDS and 1× protease inhibitor). The lysate was then pre-cleared for 1 h by mixing 400 μl sample with protein G Dynabeads (Thermo Fisher Scientific, #10004D). Afterward, the pre-cleared lysate was incubated with a primary antibody [goat anti-Myc #ab9132 or goat anti-mouse IgG (H + L) #115-005-003] overnight at 4°C. Protein G Dynabeads were then added to the samples, followed by a 3 h incubation, and washing with 1× PBS. Finally, proteins were extracted by adding 30 μl modified RIPA buffer and boiling of the sample at 95°C for 3 min. To determine whether the interactions were RNA-mediated, the lysate was incubated with 50 μg/ml RNase A at 37°C for 15 min, prior to pre-clearing. Fifty microliters of lysate was then used to extract RNA and confirm successful degradation, while the rest was used for immunoprecipitation. For the reciprocal IP, nuclear extract was isolated from untreated HEK293T cells with the NE-PER nuclear cytoplasmic extraction kit, using the manufacturer’s guidelines (Thermo Fisher Scientific, #78833). The immunoprecipitation was further performed as described above, except that the lysate was incubated with a primary antibody against U2AF1 (rabbit anti-U2AF35, #ab172614).

### Immunoblot

Immunoprecipitated samples were run on a 4–20% denaturing polyacrylamide gel and transferred onto a nitrocellulose membrane using a semi-dry transfer. Membranes were blocked in 5% milk with 0.05% Igepal and blotted with a 1:1000 dilution of primary antibodies rabbit anti-PDCD7 (#ab131258), rabbit anti-Myc (#ab9106), mouse anti-SRSF1 (Invitrogen, #32-4500), rabbit anti-U2AF1 (#ab172614), rabbit anti-U2AF2 (#ab37530), rabbit anti-SF3B1 (Cell Signaling, #14434S) and rabbit anti-SNRNP40 (Sigma, #HPA026527), followed by addition of 1:2000 dilution of secondary IRDye antibodies from Li-Cor.

For siRNA-transfected a549 cells, nuclear protein was extracted using the NE-PER nuclear cytoplasmic extraction kit (Pierce, #78833), supplemented with cOmplete Protease Inhibitor Cocktail (Roche, #11836170001) and phosSTOP phosphatase inhibitors (Roche, #4906837001) according to the manufacturer’s instructions. Thirty micrograms of protein per lane was resolved on NuPAGE Novex Bis-Tris 4–12% polyacrylamide gels and transferred unto Immobilon-FL PVDF membrane (Millipore, #05317), The membrane was blotted with 1:1000 dilution of primary antibody rabbit anti-PDCD7 (ab131258) and 1:2000 rabbit anti-TBP (ab28175) diluted in Intercept (PBS) buffer (Li-Cor, #927-70001), followed by 1:10 000 goat anti-rabbit 800 (Li-Cor, #926-32221) secondary antibody.

### Mass spectrometry

Protein samples (*N* = 6 per condition) were submitted to the Proteomics and Metabolics Core facility at the University of Connecticut for a slightly modified filter-aided sample preparation method in a Microcon YM-10 10 kDa molecular weight cutoff (MWCO) filter (Thermo Fisher Scientific) (27). Briefly, all IP elutions were diluted to 250 μl final volume using UA buffer (8 M urea in 0.1 M Tris–HCl, pH 8.5) and reduced using 25 mM dithiothreitol for 1.5 h at 37°C. The samples were added to the MWCO filter and spun at 14 000 × *g* for 40 min, washed with 200 μl UA buffer and spun again using spin condition 1 (14 000 × *g* for 40 min). Cys residues were carbamidomethylated using 50 mM iodoacetamide in UA buffer for 15 min in the dark at 37°C and then centrifuged at spin condition A. Proteins were washed using two cycles of resuspension in 100 μl UB buffer (8 M urea in 0.1 M Tris–HCl, pH 8.0) and centrifuged using spin condition 1 (14 000 × *g* for 30 min). Proteins were resuspended in 50 μl UB buffer, removed from the MWCO filter and placed into a new 1.7-ml Eppendorf safe-lock tube. The MWCO filter was washed with two aliquots of 50 μl of 0.1 M ammonium bicarbonate and pooled to result in a final urea concentration <1 M. The first stage of proteolysis was initiated by adding Endoproteinase LysC (Pierce) at a 1:50 protein:protein (w/w) ratio for 16 h at 37°C on a thermo shaker (Thermo Scientific). Sequencing Grade Modified Trypsin (Promega) was then added at a 1:50 protein:protein (w/w) ratio and allowed to proceed for an additional 8 h at 37°C. Enzymatic digestions were quenched using concentrated formic acid to result in a final pH of 2.5 and desalted using C18 Peptide Desalting Spin Columns (Pierce) following manufacturer’s instructions.

Peptide samples were subject to mass analysis using a Thermo Scientific Ultimate 3000 RSLCnano ultrahigh-performance liquid chromatography (UPLC) system coupled directly to a high-resolution Thermo Scientific Q Exactive HF mass spectrometer. An aliquot of each peptide preparation was injected onto a PepMap RSLC C18 analytical column (2 μm, 100 Å, 75 μm × 25 cm, Thermo Scientific) and subject to a 90 min, 300 nl/min reversed-phase UPLC method. Peptides were eluted directly into the Q Exactive HF using positive mode nanoflow electrospray ionization. A data-dependent top 15 tandem mass spectrometry (MS/MS) acquisition method was used that implemented the following parameters for MS scan acquisition: 60 000 resolution, 1e6 AGC target, maximum ion time of 60 ms and a 300–1800 *m*/*z* mass range. MS/MS scan acquisition included the following parameters: 15 000 resolution, maximum ion time of 40 ms, isolation window of 2.0 *m*/*z*, 30 s dynamic exclusion window, normalized collision energy of 27, a 200–2000 *m*/*z* scan range and charge exclusion ‘on’ for all unassigned, +1 and >+8 charged species.

Peptides were identified and quantified via label-free quantification using MaxQuant (v1.6.0.1) and the embedded Andromeda search engine (28). The raw data were then searched against an in-house generated protein database consisting of the myc-tagged Pdcd7 primary sequence and the entire UniProt *Homo sapiens* proteome database (identifier UP000005640, accessed 22 April 2017). The following parameters were used for the search: variable modifications, oxidation of Met, acetylation of protein N-termini and Gln to pyro-Glu conversion; fixed modifications, carbamidomethylation of Cys, trypsin enzyme specificity with up to two missed cleavages, LFQ quantitation ‘on’ and a minimum of five amino acids per peptide. All results were filtered to a 1% false discovery rate at the peptide and protein levels; all other parameters were kept at default values. MaxQuant-derived output was uploaded into Scaffold Q+S (v 4.0, Proteome Software) for visualization and further analysis.

Only proteins for which exclusive unique spectrum counts were detected in five out of the six Pdcd7 IPs were included for further analysis. Interacting proteins were defined as having a fold change in the sum of exclusive unique spectrum counts between the Pdcd7-Myc and control condition >2. To identify interacting proteins that could play a role in exon definition, all interacting proteins were submitted to DAVID for functional annotation analysis, and proteins that significantly enriched for the function mRNA splicing were extracted.

### RNA extraction, cDNA preparation and PCR analysis

#### U11-null dorsal telencephalon

Dorsal telencephalons were dissected from E12 *Rnu11*^WT/Flx^::*Emx1-Cre*^+/−^ (*N* = 3) and *Rnu11*^Flx/Flx^::*Emx1-Cre*^+/−^ (*N* = 3) embryos and individually used for RNA extraction. The tissue was triturated in 100 μl TRIzol (Thermo Fisher Scientific, #15596018) and RNA was extracted using the DirectZOL RNA Miniprep Plus Kit (Zymo Research, #R2072), as per the manufacturer’s instructions. Five hundred nanograms of total RNA was then used for cDNA synthesis, as described previously (29) and 25 ng of cDNA was used for reverse transcriptase PCR (RT-PCR) analyses.

#### HEK293T cells

For the morpholino experiments, HEK293T cells were resuspended in 1 ml TRIzol (Thermo Fisher Scientific, #15596018) and RNA was extracted using phenol:chloroform, as per manufacturer’s instructions. For RT-PCR analyses, 1 μg of total RNA was used for cDNA synthesis. For the siRNA screen, HEK293T cells were resuspended in 100 μl TRIzol and RNA was extracted using the DirectZOL RNA Microprep Kit (Zymo Research, #R2062). One hundred nanograms of total RNA was then used for cDNA synthesis and 25 ng of cDNA was used for RT-PCR analysis. To confirm downregulation of *PDCD7, RNPC3* and *ZRSR2*, we performed quantitative RT-PCR (qRT-PCR) analysis on 25 ng cDNA. The Cq values were then normalized to the expression of *GAPDH*.

#### A549 cells

Total RNA was extracted from A549 cells using TRIsure reagent (Bioline, #BIO-38033) following the manufacturer’s guidelines. One microgram of RNA was then used for cDNA synthesis using the SuperScript IV First-Strand Synthesis System (Invitrogen, #18091200) and random hexamer priming according to the manufacturer’s instructions.

#### PBMCs from individuals

PBMCs from individuals were pelleted and washed three times with 1× PBS. Afterward, the pellet was resuspended in 100 μl TRIzol and the RNA was extracted using the DirectZOL RNA Microprep Kit (Zymo Research, #R2062). One hundred nanograms of total RNA was then used for cDNA synthesis and 20 ng of cDNA was used for RT-PCR analysis. To determine the expression of minor spliceosome snRNAs, we performed qRT-PCR analysis on 25 ng cDNA. The Cq values were then normalized to the expression of *RN7SK*.

### ImageJ quantification

To determine mis-splicing index (MSI) values based on RT-PCR analysis, we employed ImageJ. Band intensity of canonically spliced and alternatively spliced products was calculated and used to determine the MSI value as was done for RNA-seq [i.e. intensity of AS product/(intensity of AS product + intensity of canonical product)].

### Bioinformatics analysis

#### Library preparation

Total RNA from the morpholino experiments and patients was depleted from ribosomal RNA using the RiboZero kit (#MRZH116) by the Center for Genome Innovation at the University of Connecticut. A cDNA library was then prepared using the Illumina TruSeq Stranded Total RNA Library Sample Prep Kit (#RS-122-2201) and sequenced on the Illumina NextSeq 500. This resulted in 60–100 million paired-end 151-bp reads per sample. RNA-seq of two individuals with Roifman syndrome and unaffected carriers had been reported previously (30). The RNA-seq from U11 cKO embryos had also been reported previously (18).

#### Gene expression analysis

Reads from each sample were aligned to the hg38 genome (UCSC Genome Browser) using Hisat2 as described previously (18,31). Gene expression was then determined by IsoEM2, followed by differential gene expression by IsoDE2, as described previously (18,32,33). Only those MIGs differentially expressed in the disease state compared to both the unrelated healthy controls and the respective unaffected carriers were included.

#### Intron retention and AS analysis

Minor intron coordinates were downloaded from the Minor Intron Database (14). Coordinates of the flanking major introns were then extracted for the canonical MIG transcripts (as defined by Ensembl). These were then used to determine retention and AS levels as described previously (14). While minor intron retention can be considered a form of AS, the pipelines used to detect retention versus aberrant exon–exon junctions are different. Therefore, in this manuscript, we discuss minor intron retention separately from other forms of AS, such as exon skipping, cryptic SS usage and cryptic exon usage. Briefly, introns were considered retained when at least one read aligned to each exon–intron boundary, with a minimum of four reads in total. Moreover, the intron coverage had to be at least 95%. AS events were considered present when at least *n* number of reads supported the novel exon–exon junction. Here, *n* is defined as the number of uniquely mapped reads for a sample divided by 3 million. The MSI value was then calculated as the number of reads supporting an aberrant splicing event/(the number reads supporting an aberrant splicing event + the number of reads supporting the canonical splice junction). Additionally, the %MSI_AS_ for AS events had to be at least 10% to be included in downstream analyses. For Lowry–Wood syndrome, elevated AS and retention were then determined by a ≥2-fold change in %MSI in the patient compared to the unaffected carrier and the average of the healthy controls. Elevated retention and AS around minor introns in the other datasets were determined by either performing a one-way ANOVA, followed by the post-hoc Tukey test, or a Welch's *t*-test on the %MSI values.

#### Functional enrichment analysis

Genes were submitted for functional enrichment analysis to DAVID (34). Only significant GO terms (Benjamini–Hochberg adjusted *P*-value <0.05) were reported.

#### Consensus sequence analysis

Novel junction coordinates were extracted for each upregulated AS event in the U11 cKO dorsal telencephalon using BEDTools (35). These were then utilized to extract the novel intronic sequences generated by AS events with the BEDTools getfasta tool. Finally, frequency plots of the annotated and novel SSs were made using WebLogo (36). If not a single novel exon–exon junction was supported by >10 reads, this MIG was excluded from the analysis.

#### ORF analysis

To determine the effect of AS events across minor introns on the open-reading frame (ORF), known exon–exon junctions for the canonical MIG transcripts were adjusted to contain the novel junction coordinates using BEDTools (35). These were then used to generate the full coding sequence as well as for *in silico* translation. To predict whether an alternatively spliced transcript with premature stop codon would be targeted for NMD, the location of the stop codon was compared to the annotated last exon–exon junction of each gene. If the stop codon was >50 nt upstream of the last exon–exon junction, the transcript was predicted to be targeted to NMD, otherwise it was considered to be translated into protein (37). The effect of AS on protein domains was determined using the pfam database (38). Alternatively spliced MIGs that did not have a single novel exon–exon junction supported by >10 reads were excluded.

#### Principal component analysis

Principal component analysis (PCA) was performed based on MIG expression (TPM values), retention levels (%MSI_Ret_) and AS levels (%MSI_AS_) using the default settings in ClustVis (39).

### Quantification and statistical analysis

Statistical details of the experiments can be found in the figures and figure legends, as well as the ‘Results’ section. The statistical tests used to identify minor introns with elevated retention or AS are described in the ‘Bioinformatics Analysis’ section. Statistical differences in %MSI_AS_ as calculated by ImageJ were determined by one-way ANOVA, followed by the post-hoc Tukey test. Correlation between %MSI_AS_ values determined by RNA-seq and ImageJ analysis was established using a Pearson’s correlation coefficient test. To determine whether there were statistically significant differences in intronic features between minor introns that were alternatively spliced and not, we performed a Kruskal–Wallis test, followed by the post-hoc Dunn test. Significant differences in expression levels of PDCD7, RNPC3 and ZRSR2 were determined by Welch’s *t*-test. *P* < 0.05 was considered as significant in all analyses.

## RESULTS

### Inhibition of each minor spliceosome snRNA results in elevated alternative splicing around minor introns

According to the exon-definition model, splicing of major introns positioned immediately upstream of a minor intron would require the major and minor spliceosomes to interact with each other. If these exon-bridging interactions were to be disrupted, the 3′ end of the major intron would not be properly identified and the major spliceosome would instead utilize a major-type BPS further downstream. In other words, disruption of these exon-definition interactions would result in the major spliceosome executing AS, specifically skipping the exons flanking the minor intron. Based on the components that are required for the identification of major introns, we hypothesized that U11 and U12 snRNPs would be the equivalent components important for exon-definition interactions involving minor introns. To test this hypothesis, we sought to inhibit the different minor spliceosome components and determine the effect on splicing of minor introns. To this end, we employed antisense MOs to inhibit U12, U4atac and U6atac snRNAs individually in HEK293 cells, followed by capture of the nascent RNA and RNA-seq (Supplementary Figure S1A). While this strategy would reduce the number of MIGs for analysis, it allowed us to capture the most immediate splicing defects after minor spliceosome inhibition. To determine the efficiency of minor spliceosome inhibition, we first analyzed the level of minor intron retention. Compared to the control morpholino, we found that inhibition of U12 snRNA led to significantly elevated retention of 22 minor introns, whereas inhibition of U4atac and U6atac snRNAs both resulted in the significant retention of 74 minor introns (Supplementary Figure S1B–D; Dataset S1). Importantly, a morpholino against U2 snRNA, a major spliceosome component, did not result in elevated minor intron retention, and only led to the enhanced splicing of a minor intron in *DERL3* (Supplementary Figure S1E).

**Figure 1.**
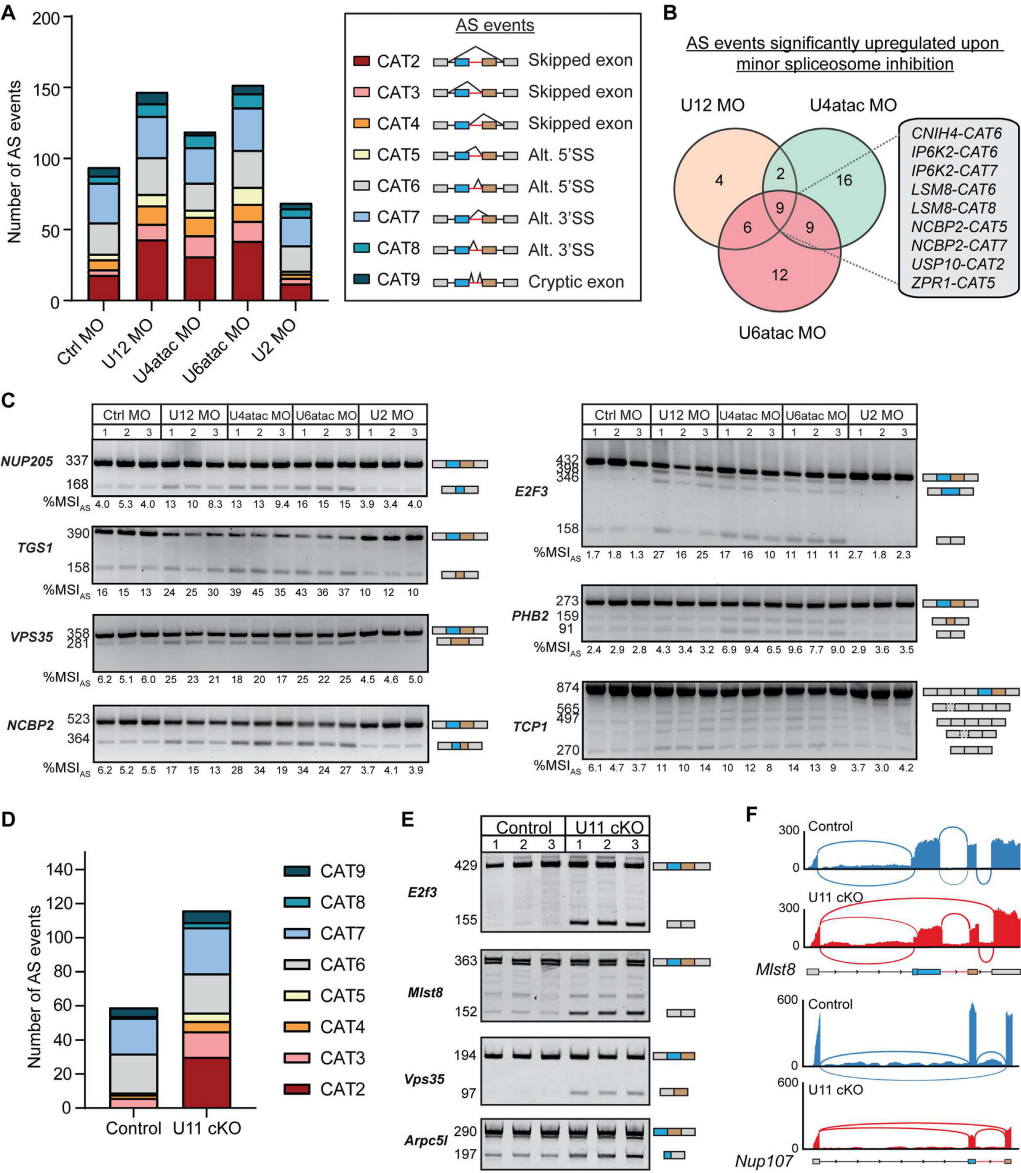
Inhibition of each minor spliceosome snRNA results in elevated AS around minor introns. **(A)** Bar graph with number of AS events around the minor introns in each morpholino (MO) condition. The different types of AS corresponding to the color codes are shown on the right. The investigated AS events are also schematized on the right, where the minor intron is colored red and the flanking upstream and downstream exons are colored blue and brown, respectively. CAT = category; Alt = alternative; SS = splice site. **(B)** Venn diagram showing the overlap of minor introns with significant upregulation of AS upon inhibition of the minor spliceosome. The AS events that are significantly upregulated in all three conditions are shown on the right. **(C)** Agarose gel images of RT-PCR products resulting from AS around minor introns. Product size (in base pairs) of Sanger-sequenced products is shown on the left; product schematics are shown on the right. The %MSI_AS_ was calculated using ImageJ. **(D)** Bar graph with number of AS events around the minor intron in U11 cKO embryos. **(E)** Acrylamide gel images of RT-PCR products resulting from AS around minor introns. Product size (in base pairs) of Sanger-sequenced products is shown on the left; product schematics are shown on the right. **(F)** Sashimi plots showing combined AS usage around minor introns. See also Supplementary Figures S1–S3.

Next, we employed our customized bioinformatics pipeline to detect *de novo* AS across minor introns in the aforementioned conditions (14). Spliced reads around the minor introns were used to determine the level of eight different types of AS across the samples, including exon skipping, cryptic 5′SS usage and cryptic 3′SS usage (Figure 1A, right). This level of AS was then represented by a %MSI_AS_ value ranging between 0 and 100%. Using this approach, we found 94 AS events around minor introns in the control condition (Figure 1A). This was increased upon inhibition of U12 snRNA, U4atac snRNA and U6atac snRNA, where we detected 147, 119 and 152 AS events, respectively (Figure 1A). In contrast, inhibition of U2 snRNA resulted in a reduction of AS around minor introns (69 AS events) (Figure 1A). The increased AS levels upon inhibition of the minor spliceosome components were primarily due to an increase in exon skipping (CAT2–CAT4) (Figure 1A; Dataset S2). While also present in the control condition, the usage of this type of AS event had doubled upon minor spliceosome inhibition (Figure 1A). This finding is in keeping with a model whereby the minor spliceosome engages in exon-definition interactions. Overall, 58 AS events were significantly upregulated when one or more minor spliceosomal components were inhibited (Dataset S2). Together, 60% of these events involved the skipping of one or both exons flanking the minor intron, whereas cryptic exon usage was not affected at all (Supplementary Figure S2A). Intersection analysis of the 58 significantly upregulated AS events revealed that only 9 (15%) were common to inhibition of U12, U4atac and U6atac snRNAs, such that the splicing of 6 minor introns was affected (Figure 1B; Dataset S2). Instead, AS across the majority of minor introns was significantly upregulated in just one or two of the morpholino conditions (Figure 1B; Dataset S2). While these differences might in part be explained by variation in the efficiency of morpholinos to inhibit each snRNA, they also point to a target specificity of each minor spliceosome component. The significantly upregulated AS events were then further confirmed by RT-PCR analysis on total RNA and Sanger sequencing (Figure 1C). Importantly, quantification of the RT-PCR products using ImageJ revealed a statistically significant, moderate to high, positive correlation with the %MSI_AS_ values obtained by RNA-seq (Supplementary Figure S2B). While inhibition of U12, U4atac and U6atac snRNAs all resulted in upregulation of AS around minor introns, the levels at which AS occurred in each MIG varied depending on the snRNA that was inhibited. For example, AS around the minor intron of *PHB2* was most highly upregulated upon inhibition of U4atac and U6atac snRNAs, whereas AS around the minor intron of *E2F3* was most responsive to inhibition of U12 snRNA (Figure 1C; Supplementary Figure S2C). Thus, the inhibition of U12, U4atac and U6atac snRNAs differentially affects AS across minor introns, in a gene-specific manner. Nonetheless, inhibition of all three minor spliceosome snRNAs resulted in elevated exon skipping in a subset of MIGs.

**Figure 2.**
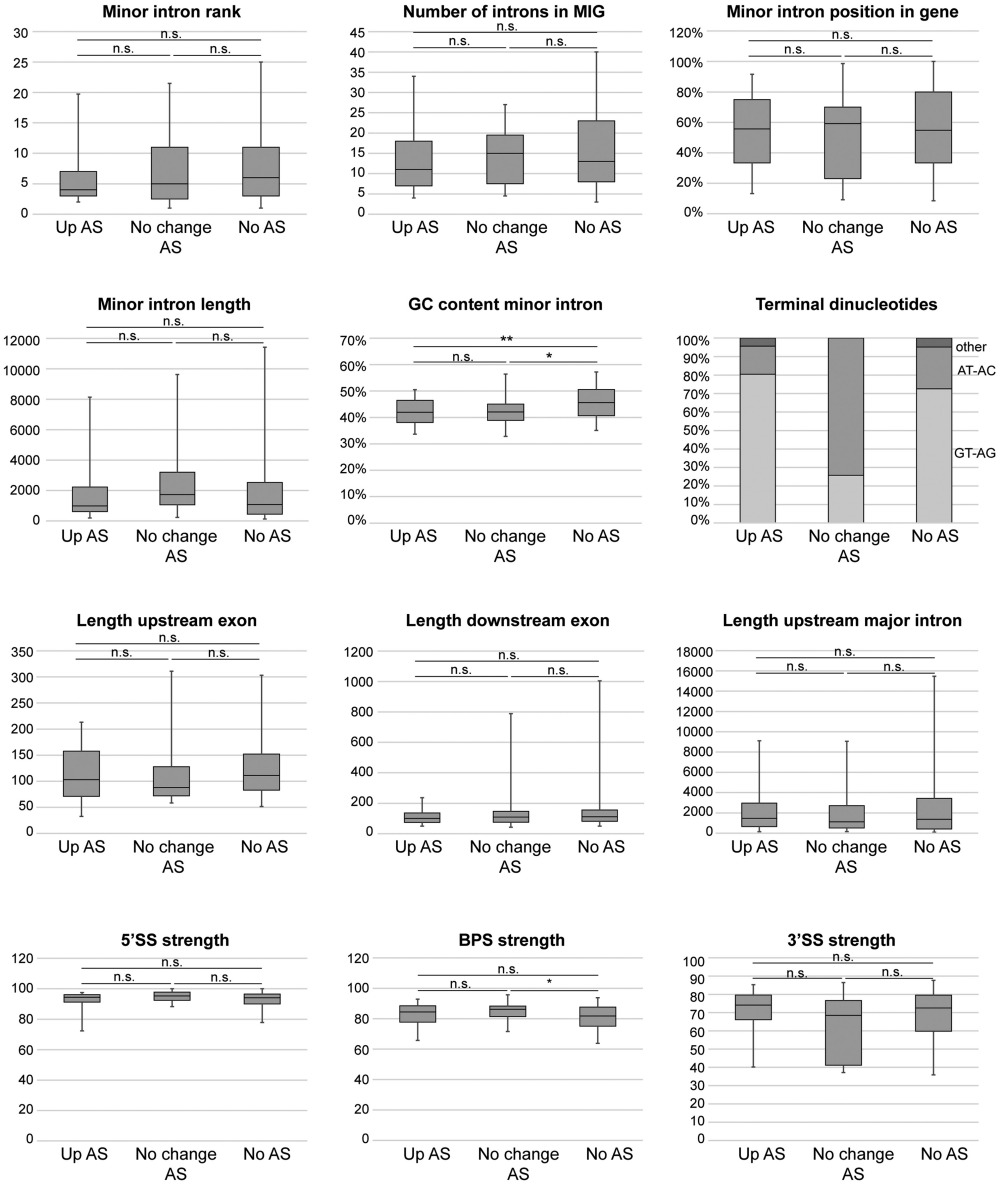
Alternatively spliced minor introns have a lower GC content than canonically spliced minor introns. Boxplots showing the 5th to 95th percentiles of several intronic and gene features for minor introns that show elevated AS upon U11 loss (up AS; 46 introns), no change in AS upon U11 loss (no change AS; 31 introns) or are not alternatively spliced at all (no AS; 631 introns). Statistical significance was determined by the Kruskal–Wallis test, followed by the post-hoc Dunn test. n.s. = not significant; *P < 0.05; **P < 0.01.

Since morpholinos have not been proven effective against U11 snRNA, we studied the effect of U11 snRNA inhibition of AS of minor introns by analyzing RNA-seq from the dorsal telencephalon of E12 U11 cKO embryos (5,18). Here, we detected a total of 117 AS events around minor introns in either the control or U11 cKO (Figure 1D; Dataset S3). Of these, 58 AS events (49.6%) were significantly upregulated in the U11 cKO (Supplementary Figure S3A). Particularly, 29 out of the 30 AS events that involved skipping of both exons flanking the minor intron (CAT2) were significantly elevated upon U11 loss (Supplementary Figure S3A). The exclusive use of these AS events in the U11 cKO was also validated by RT-PCR and Sanger sequencing (Figure 1E and F; Supplementary Figure S3A and B; Dataset S3). Thus, our data show that inhibition of all minor spliceosome snRNAs results in the production of aberrant MIG transcripts, especially through elevated levels of exon skipping. Nevertheless, not all minor introns were alternatively spliced in response to minor spliceosome inhibition. Therefore, we next aimed to identify the features that made some minor introns susceptible to AS. To this end, we analyzed intronic characteristics that have previously been shown to affect the AS status of major introns for three groups of introns: (1) minor introns with significantly elevated AS in the U11 cKO; (2) minor introns that were alternatively spliced in both the control and U11 cKO; and (3) minor introns that were not alternatively spliced in either condition (40–42). We found no statistical difference in the position of the minor intron within the MIG, the total number of introns within the MIG, or the length of the minor intron, the length of the upstream and downstream exons, or the length of the upstream major intron between these three groups (Figure 2). While we did observe a significant difference in the GC content of minor introns that were alternatively spliced in the control and/or U11 cKO versus those that were not, this did not explain why some minor introns showed elevated AS upon minor spliceosome inhibition (Figure 2). Similarly, we detected a significant difference in the BPS strength of minor introns that were alternatively spliced in the control and U11 cKO versus those that were never alternatively spliced (Figure 2). However, we did not observe a significant difference in the strength of the 5′SS or 3′SS between minor introns that were and were not alternatively spliced upon minor spliceosome inhibition (Figure 2). Finally, using DREME, we were unable to find a specific motif in the minor introns that were affected by U11 loss (43). Thus, these data suggest that either a combination of these features or an additional layer of regulation might explain why only a subset of minor introns is affected by minor spliceosome inhibition.

### Minor spliceosome protein PDCD7 (U11-59K) interacts with the major spliceosome

The increase in exon skipping upon minor spliceosome inhibition suggests that introns in at least a subset of MIGs are spliced according to the exon-definition model. After all, this model postulates that disruption of exon-definition interactions would result in the utilization of a major-type BPS/3′SS further downstream by the major spliceosome. Indeed, when we analyzed the consensus sequences of the newly utilized 5′SS and 3′SS of alternatively spliced minor introns in the U11 cKO, these resembled known major-type consensus sequences (Supplementary Figure S4A). These findings support the hypothesis that the minor and major spliceosomes normally interact to regulate the canonical splicing of minor introns and flanking major introns.

**Figure 3.**
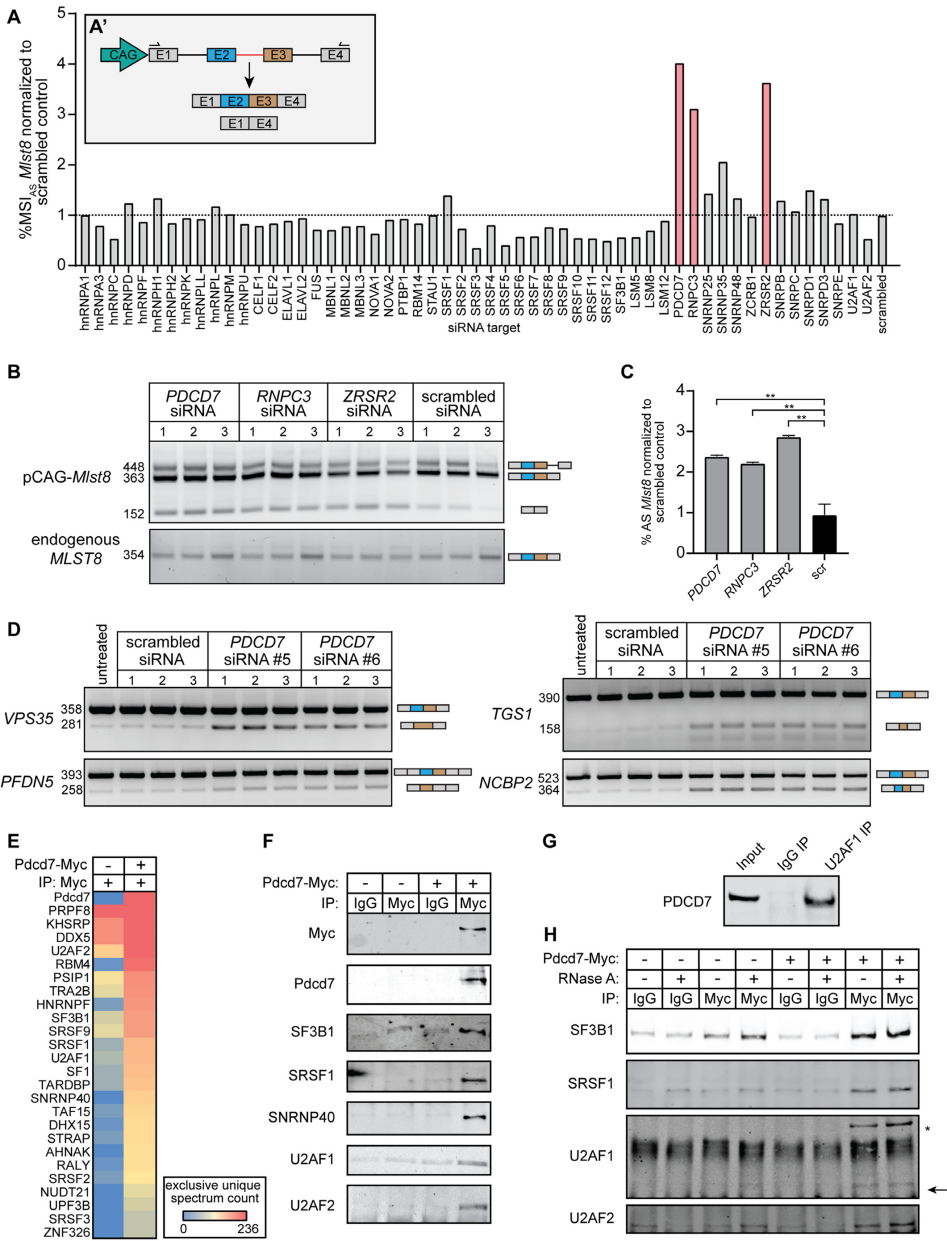
U11-59K (PDCD7) mediates exon-definition interactions between the major and minor spliceosomes. **(A)** Bar graph showing the %MSI_AS_ of the *Mlst8* splicing reporter, in response to siRNA against splicing factors. Inset shows schematic of the *Mlst8* splicing reporter and the resulting products. Agarose gel image of the *Mlst8* splicing reporter RT-PCR products resulting from downregulation of minor spliceosome proteins **(B)** and quantification of the %MSI_AS_ using ImageJ **(C)**. Significance was determined using one-way ANOVA, followed by the post-hoc Tukey test. **(D)** Agarose gel images of RT-PCR products resulting from AS around minor introns upon knockdown of PDCD7 in A549 cells. **(E)** Heatmap of the exclusive unique spectrum count of the Pdcd7-interacting proteins involved in splicing. **(F)** Immunoblot for major spliceosome proteins that immunoprecipitated with Pdcd7. **(G)** Immunoblot for PDCD7 after immunoprecipitation of endogenous U2AF1. **(H)** Immunoblot for major spliceosome proteins that immunoprecipitated with Pdcd7 with and without RNase A treatment. Arrow points at predicted molecular weight of U2AF1. Asterisk marks predicted molecular weight of U2AF2. *P < 0.05; **P < 0.01. See also Supplementary Figures S4–S6.

For genes consisting exclusively of major introns, it is known that U1-70K is crucial for the maintenance of the exon-bridging interactions (44). However, the minor spliceosome does not contain U1-70K, nor the U2AF complex, and as such the proteins involved in exon-definition complexes between major and minor introns in MIGs remain unidentified (45). Therefore, we next designed an AS mini-gene construct with a minor intron and the flanking major introns of the MIG *Mlst8*, such that disruption of the interactions between major and minor spliceosomes would result in the elevated production of an alternatively spliced transcript consisting of exons 1 and 4 (Figure 3A). We then leveraged this readout to perform an siRNA screen against 55 known splicing and AS factors, followed by RT-PCR analysis for the Mlst8 construct (Supplementary Figure S4B). Quantification of the alternatively spliced product as a ratio of the canonically spliced transcript (%MSI_AS_) showed that the largest increase in exon skipping resulted from siRNAs against *PDCD7* (U11-59K), *RNPC3* (U11/U12-65K) and *ZRSR2* (Urp) (Figure 3A; Supplementary Figure S4C). After successful confirmation of downregulation of these three genes, we repeated the screen with siRNAs against *PDCD7*, *RNPC3* and *ZRSR2* in triplicate (Figure 3B and C; Supplementary Figure S5A and B). This revealed that skipping of the exons flanking the minor intron of *Mlst8* was indeed significantly >2-fold upregulated upon inhibition of these splicing factors, compared to the scrambled siRNA (Figure 3B and C). Thus, the cross-talk between the major and minor spliceosomes might directly involve protein components of the minor spliceosome.

We decided to further explore the role of PDCD7 (U11-59K), because it is the only U11 snRNP component that showed increased exon skipping upon knockdown (Figure 3B and C). First, we wanted to test whether downregulation of PDCD7 would also impact the splicing of those endogenous MIGs that were affected in the morpholino experiments (Figure 1A–C). In agreement with the splicing reporter results, RT-PCR for these MIGs revealed an increase in AS, albeit modest (Supplementary Figure S5C). This finding was not surprising, since we have previously reported that minor intron splicing is differentially regulated across cell types (14). Therefore, we next wanted to see whether downregulation of PDCD7 in another cell line would show more robust AS. To this end, we knocked down PDCD7 in the lung adenocarcinoma A549 cells and performed RT-PCR analysis, which indeed revealed a more robust increase in AS, including elevated exon skipping (Figure 3D; Supplementary Figure S5D and E).

These findings led us to hypothesize that PDCD7 could perform an analogous role to U1-70K in the maintenance of exon-definition interactions. To test this, we sought to identify Pdcd7-interacting proteins by transfecting HEK293 cells with pCMV-Pdcd7-Myc, followed by immunoprecipitation and mass spectrometry. To determine the most robust interacting partners, we curated proteins with ≥2-fold higher spectrum counts in the Pdcd7-Myc condition compared to a negative control, which resulted in a list of 175 proteins (Supplementary Figure S6). Since we wanted to study exon-bridging interactions, we then focused on proteins with a known role in splicing. This resulted in a list of 25 proteins, of which many are known to interact with each other (Figure 3E). Specifically, we detected several proteins that have been identified in the major spliceosome E and A complex, such as SF1, SRSF1, SRSF2, SRSF3, SF3B1, U2AF1 and U2AF2, which were validated by western blot (Figure 3E and F) (46,47). Moreover, the U5 snRNP components SNRNP40 and PRPF8 co-immunoprecipitated with Pdcd7 (Figure 3E and F) (48). Importantly, immunoprecipitation of endogenous U2AF1 also pulled down endogenous PDCD7, further supporting our finding that the major and minor spliceosomes can interact through PDCD7 (Figure 3G). Finally, to test whether these interactions were direct protein–protein interactions, or whether they were RNA-mediated, we performed IP for Pdcd7, followed by RNase A treatment and western blotting. Again, this revealed that Pdcd7 interacted with major spliceosome proteins such as U2AF1, U2AF2 and SF3B1, suggesting these are direct protein–protein interactions (Figure 3H). In all, these data suggest that U11 snRNP normally interacts with the major spliceosome E or A complex through PDCD7. Maintenance of these interactions is crucial for the proper splicing of minor introns and the flanking major introns, and when disrupted can result in the production of aberrant MIG transcripts.

### Alternatively spliced MIG transcripts are not subjected to NMD but bound to polysomes

**Figure 4.**
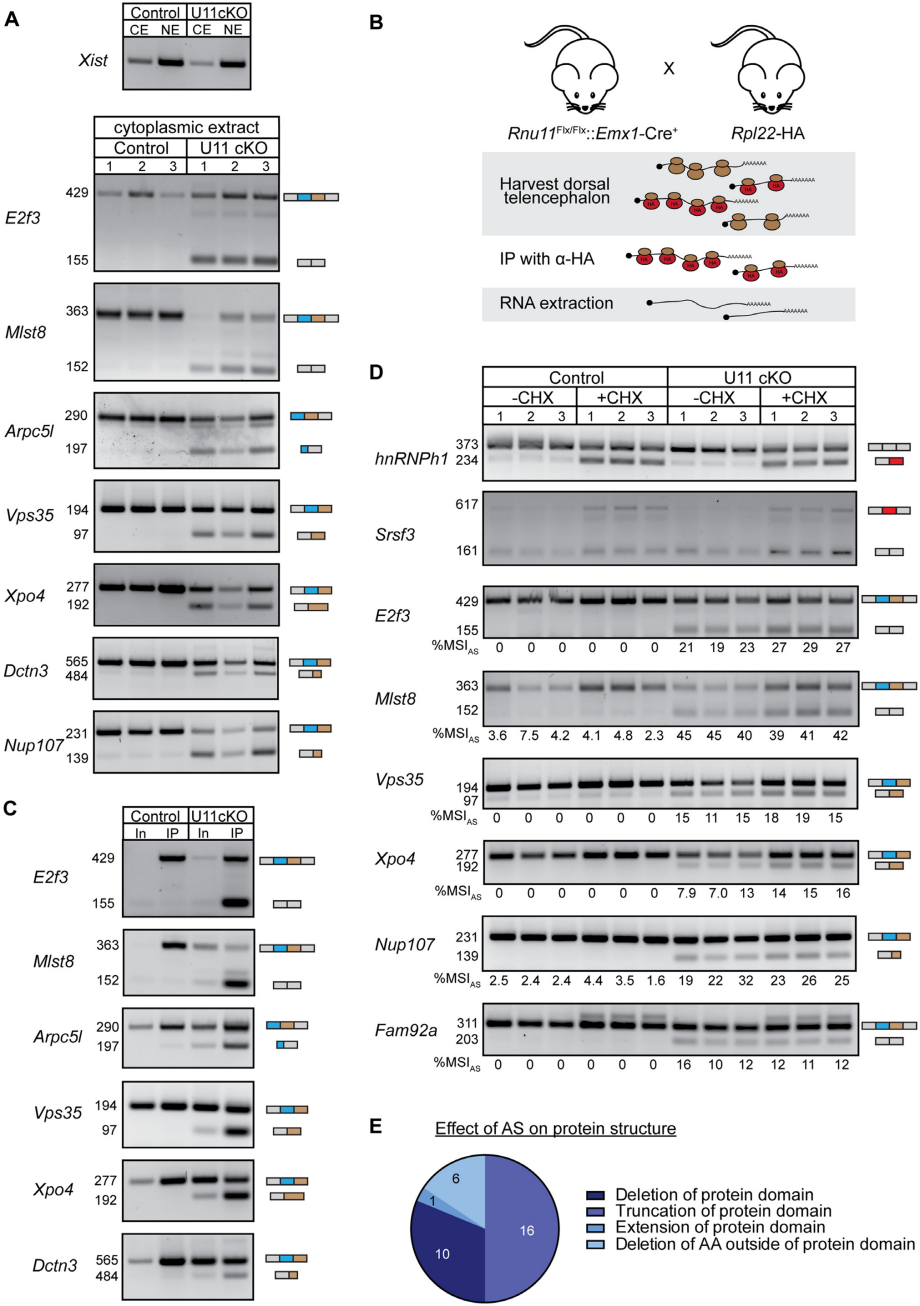
Alternatively spliced MIG transcripts are not subjected to NMD but bound to polysomes. **(A)** Agarose gel images showing the nuclear lncRNA *Xist* as a fractionation control; shown below are alternatively spliced RT-PCR products in the cytoplasmic fraction of the E12 dorsal telencephalon. **(B)** Schematic of experimental design to isolate RNA bound to polysomes in the U11 cKO Ribotag mouse. **(C)** Agarose gel images of alternatively spliced RT-PCR products in whole extract (In, input) and bound to polysomes (IP) of E14 control and U11 cKO dorsal telencephalons. **(D)** Agarose gel images of alternatively spliced RT-PCR products in E12 control and U11 cKO cultured telencephalons with and without NMD inhibitor cycloheximide. The %MSI_AS_ was calculated using ImageJ. **(E)** Pie chart with the effect of AS on protein domains of affected MIGs. AA = amino acid; CE = cytoplasmic extract; NE = nuclear extract; CHX = cycloheximide. See also Supplementary Figure S7.

Aberrant transcripts are normally quickly detected by quality control mechanisms, resulting in their degradation by the nuclear exosome or NMD (49). To test whether alternatively spliced MIG transcripts would be subject to exosome-mediated degradation in the nucleus, we fractionated the dorsal telencephalons from control and U11 cKO E12 embryos into a nuclear and cytoplasmic (CE) fraction (Figure 4A). We found that all of the AS events detected in whole-cell extract were also detected in the CE of U11 cKO embryos (Figures 1E and 4A). The successful export of these alternatively spliced MIG transcripts led us to investigate the effect of AS on the open reading frame (ORF), as frameshifts and premature stop codons are predicted to activate NMD (50). We found that 47% of the AS events were in frame, whereas the other half resulted in a premature stop codon. In 83% of the cases, this premature stop codon was also predicted to activate the NMD pathways, whereas for five MIGs the premature stop codon was positioned close enough to the last exon–exon junction that it was not predicted to trigger the NMD pathway.

To explore whether a subset of the alternatively spliced MIG transcripts was indeed translated, we next crossed our U11 cKO mice to Ribotag mice (20). These mice express an HA-tagged version of the ribosomal protein *Rpl22* in the cells that express Cre recombinase. As such, polysomes of all U11-null cells can be purified by immunoprecipitation with an anti-HA antibody. RT-PCR analysis on RNA extracted from immunoprecipitated polysomes showed the presence of all interrogated alternatively spliced MIG transcripts in the E14 U11 cKO embryos (Figure 4B and C). Even MIG transcripts that were predicted to undergo NMD by bioinformatics analyses, such as *E2f3*, *Mlst8* or *Vps35*, were bound to polysomes (Figure 4C). This finding suggested that these aberrant MIG transcripts were not subjected to NMD. To further confirm this, we next dissected the telencephalon of E12 control and U11 cKO embryos, and cultured them for 2 h in medium supplemented with cycloheximide, a known NMD inhibitor (51). In this case, the transcripts that are subject to NMD would be stabilized after addition of cycloheximide and detected at elevated levels (51). Successful inhibition of NMD in the cycloheximide-treated samples was confirmed by an increase of the alternatively spliced products of *hnRNPh1* and *Srsf3* that contain a premature stop codon and are normally degraded through the NMD pathway (Figure 4D) (52). Therefore, we next performed RT-PCR analysis for several MIGs to test whether they too were NMD targets. While the alternatively spliced MIG transcripts were upregulated in the U11 cKO compared to the control telencephalons, we observed no significant change in %MSI_AS_ for most MIGs between the telencephalons cultured in cycloheximide and those that were not (Figure 4D). Thus, although almost half of the alternatively spliced MIG transcripts were bioinformatically predicted to be NMD targets, they were in fact not subjected to NMD, and instead likely translated. Therefore, we next determined the effect of the AS on protein structure, by translating the ORF *in silico*. This revealed that the majority of AS events would result in a truncation of the protein (Figure 4E). Notably, 50% of the AS events would result in a truncation of protein domains, whereas 31% of the AS events would result in the removal of one or more entire protein domains (Figure 4E; Supplementary Figure S7A). To gain insight into the biological processes that might be affected by the production of these aberrant MIG proteins, we next performed functional annotation analysis. This showed a significant enrichment of the GO term condensed chromosome kinetochore, suggesting that cell cycle may be affected (Supplementary Figure S7B). Indeed, *Ahctf1*, *Spc24*, *Nup107* and *Dctn3*, the four alternatively spliced MIGs that enriched for this function, play known roles during mitosis (Supplementary Figure S7B and C) (53–56). Previously, we have shown a prolonged pro-metaphase to metaphase transition of U11-null radial glial cells (18). Moreover, we found that loss of U11 predominately affected the survival of self-amplifying radial glial cells (18). Thus, the loss of functional MIG-encoded proteins due to AS or a toxic gain of function acquired by aberrant MIG isoforms might have contributed to these phenotypes.

### Minor intron retention occurs more frequently in Roifman syndrome than Lowry–Wood syndrome

While our experiments in cell culture and the U11 cKO mouse revealed the presence of novel MIG isoforms upon minor spliceosome inhibition, the physiological relevance of these alternatively spliced transcripts remained unclear (Figure 1). Therefore, we wanted to test whether they could play a role in disease pathogenesis, specifically those diseases associated with mutations in minor spliceosome components. To date, five developmental diseases have been linked to germline mutations in minor spliceosome components (57). These include microcephalic osteodysplastic primordial dwarfism type 1 (MOPD1), Roifman syndrome and Lowry–Wood syndrome, which are all caused by mutation in *RNU4ATAC*, the gene that encodes U4atac snRNA (30,58–60). Moreover, mutations in *RNU12* have been linked to an early-onset form of cerebellar ataxia (24) and mutations in the minor spliceosome-specific protein *RNPC3* have been linked to isolated growth hormone deficiency (61). Based on the exon-definition model, mutations in *RNU12* and *RNPC3* have been predicted to result in elevated AS around minor introns, whereas mutations in *RNU4ATAC* were thought to solely result in elevated minor intron retention (62). Indeed, transcriptomic analysis of individuals with MOPD1 and Roifman syndrome has revealed widespread minor intron retention, but only minimal AS around minor introns (63). However, since we found that morpholinos against U4atac snRNA did result in increased exon skipping, we wondered whether AS around minor introns in these patients had not previously been observed due to differences in bioinformatics analyses (Figure 1A and B). Moreover, transcriptomic analysis of individuals with Lowry–Wood syndrome had not been reported. Therefore, we here performed transcriptomic analysis of PBMCs from individuals with Roifman syndrome and an individual with Lowry–Wood syndrome, as well as their unaffected heterozygous fathers and unrelated healthy controls, using our bioinformatics pipeline. The individual with Lowry–Wood syndrome was a 28-year-old male and a compound heterozygote for the *RNU4ATAC* variants n.120T>G and n.114G>C (Supplementary Figure S8A) (21,22). The individuals with Roifman syndrome included a 6-month-old male with two mutations *in trans* at n.17G>A and n.116A>G, and two unrelated adult males previously described in (30) (Supplementary Figure S8A).

**Figure 5.**
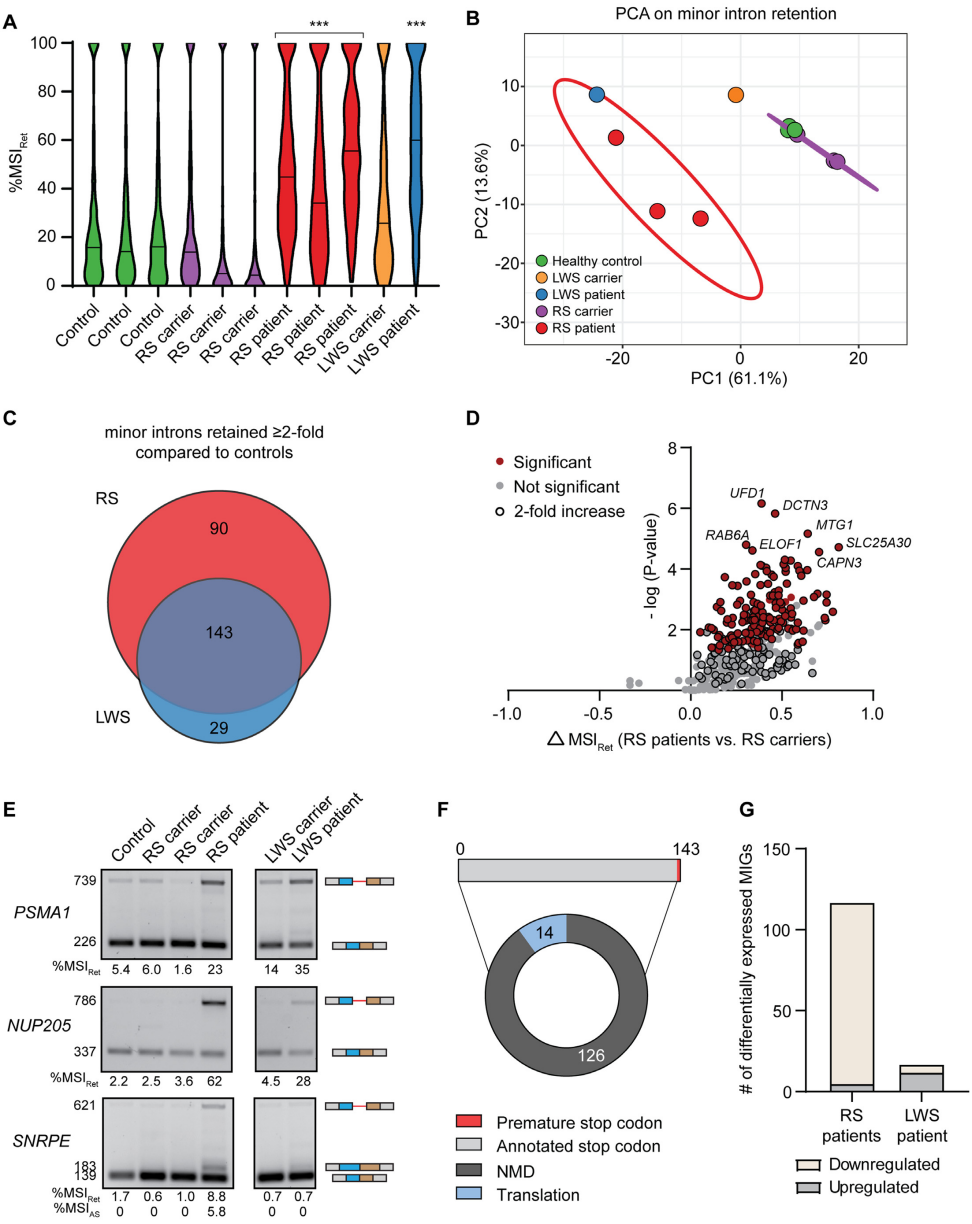
Minor intron retention occurs more frequently in Roifman syndrome than Lowry–Wood syndrome. **(A)** Violin plots of the %MSI_Ret_ for all retained minor introns in each individual. Solid line denotes median. Statistical significance was determined by the Kruskal–Wallis test, followed by the post-hoc Dunn test. ***P < 0.001. **(B)** PCA on %MSI_Ret_ values in patients and controls. Prediction ellipse is drawn such that the probability is 95% that a new observation from the same condition falls inside the ellipse. **(C)** Venn diagram of the number of minor introns with elevated retention in the RS and LWS patients compared to healthy controls and the respective carriers. **(D)** Volcano plot of MIGs with minor intron retention in RS. **(E)** Agarose gel images of RT-PCR products resulting from minor intron retention in PBMCs. Product size (in base pairs) of products is shown on the left; product schematics are shown on the right. The %MSI_Ret_ was calculated using ImageJ. **(F)** Pie chart with effect of minor intron retention on the ORF and fate of MIG transcripts. **(G)** Bar graph with number of differentially expressed MIGs in the RS patient and LWS patients compared to healthy control. RS = Roifman syndrome; LWS = Lowry–Wood syndrome; NMD = nonsense-mediated decay. See also Supplementary Figure S8.

Since minor intron retention had not previously been evaluated in Lowry–Wood syndrome, we first analyzed the minor intron retention levels (%MSI_Ret_) in all individuals. This revealed that the median %MSI_Ret_ was significantly elevated in the individual with Lowry–Wood syndrome (60%) compared to the unaffected carrier (26%) and the unrelated healthy controls (*P<* 0.0001). Moreover, minor intron retention was significantly elevated in individuals with Roifman syndrome (median: 45%, 34% and 56%) compared to the unaffected carriers (median: 14%, 5% and 4%) and unrelated healthy controls (median: 16%, 14% and 16%) (*P* < 0.0001) (Figure 5A). Consistently, PCA of the %MSI_Ret_ values partitioned the patient samples from the healthy samples (PC1: 61% of the variance) (Figure 5B). Thus, minor intron splicing is affected in both individuals with Roifman syndrome and those with Lowry–Wood syndrome.

Next, we wanted to determine which minor introns were affected upon inhibition of *RNU4ATAC*. Since we only had access to PBMCs of one individual with Lowry**–**Wood syndrome, we could not perform statistical analyses to identify minor introns with elevated retention. Instead, we extracted minor introns with a %MSI_Ret_ that was at least 2-fold higher in the patients compared to their respective carriers and the healthy controls. Using this approach, we identified 233 minor introns in 225 MIGs with elevated retention in the individuals with Roifman syndrome, and 172 minor introns in 169 MIGs with elevated retention in the individual with Lowry–Wood syndrome (Figure 5C; Dataset S4). These sets of introns contained a large degree of overlap, as 83% of all minor introns with elevated retention in the individual with Lowry–Wood syndrome were also retained at elevated levels in the individual with Roifman syndrome (Figure 5C; Dataset S4). Conversely, 61% of all minor introns with increased minor intron retention in the individual with Roifman syndrome were also retained at elevated levels in the individual with Lowry–Wood syndrome (Figure 5C; Dataset S4). Statistical analysis then revealed that the ≥2-fold increase in retention levels was significant for 63% of the minor introns in the individuals with Roifman syndrome (Figure 5D). In all, these data suggest that minor intron retention may be more widespread in individuals with Roifman syndrome than individuals with Lowry–Wood syndrome. This was visualized in Sashimi plots and corroborated by RT-PCR analysis, which revealed elevated minor intron retention in *PSMA1* and *NUP205* in both Roifman syndrome and Lowry–Wood syndrome, while minor intron retention in *SNRPE* was restricted to Roifman syndrome (Figure 5E; Supplementary Figure S8B). To test whether the difference in minor intron retention levels between Roifman syndrome and Lowry–Wood syndrome could be explained by the expression levels of U4atac snRNA, we next performed qRT-PCR for the unique snRNA components of the minor spliceosome. This revealed that U11, U12 and U4atac snRNAs were ∼2-fold upregulated in Roifman syndrome and Lowry–Wood syndrome compared to their respective carriers and healthy controls, whereas U6atac snRNA was unchanged (Supplementary Figure S8C). This upregulation of U11, U12 and U4atac snRNAs likely reflects a compensatory change in response to a non-functional minor spliceosome. Importantly, the results suggest that differences in U4atac snRNA levels do not account for the increased minor intron retention in Roifman syndrome compared to Lowry–Wood syndrome.

Retention of introns often results in the introduction of a premature stop codon, which is generally predicted to result in the degradation of the transcript through NMD (49). However, our findings in the U11 cKO suggested that not all premature stop codons result in downregulation of the transcript (Figure 4D). Of the 143 minor introns that were retained at elevated levels in both individuals with Roifman syndrome and those with Lowry–Wood syndrome, we found that retention of 140 minor introns resulted in a premature stop codon (Figure 5F). In 90% of the cases, the premature stop codon was also predicted to activate the NMD pathway, which should result in the degradation of the 124 MIG transcripts that contained these retained introns (Figure 5F). Regardless, differential expression analysis of MIGs in patients and controls revealed that only 19 of the 124 MIGs with elevated retention were significantly downregulated in Roifman syndrome and only 1 was significantly downregulated in Lowry–Wood syndrome. Thus, these data support our results that aberrant MIG transcripts are generally not subject to NMD (Figure 4D). Differential expression analysis of all MIGs revealed that 113 MIGs expressed above one TPM were significantly downregulated in Roifman syndrome, and 4 MIGs were significantly upregulated (Figure 5G; Dataset S5). Moreover, in total, 6 MIGs were significantly downregulated in Lowry–Wood syndrome, and 11 were significantly upregulated (Figure 5G; Dataset S5). Thus, minor intron retention does not generally result in the downregulation of MIGs, which is consistent with previous reports where the minor spliceosome was inhibited (18,30,63,64). Finally, PCA on MIG expression did not separate the patients from the controls on the first or second axis, which suggests that minor intron retention is a better predictor of minor spliceosome disease than MIG expression (Supplementary Figure S8D).

### Roifman syndrome and early-onset cerebellar ataxia are characterized by higher levels of AS around minor introns than Lowry–Wood syndrome

**Figure 6.**
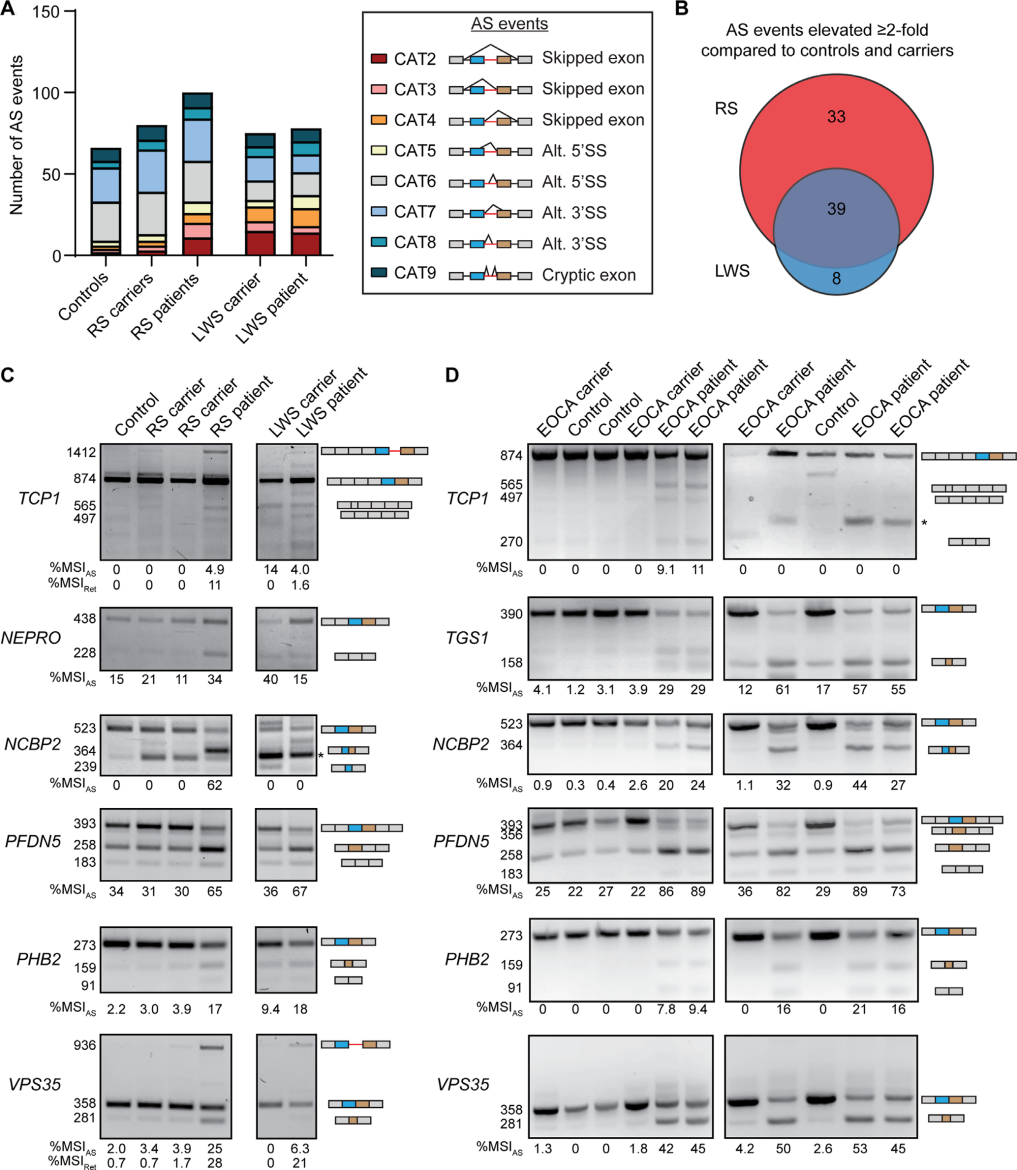
Roifman syndrome and early-onset cerebellar ataxia are characterized by higher levels of AS around minor introns than Lowry–Wood syndrome. **(A)** Bar graph with number of AS events around the minor intron in each individual. Schematics of different types of AS events are shown on the right. **(B)** Venn diagram of the number of minor introns with elevated AS in the RS and LWS patients compared to healthy controls and the respective carriers. Agarose gel images of RT-PCR products resulting from AS around minor introns in PBMCs of individuals with mutation in *RNU4ATAC***(C)** and *RNU12***(D)**. Product size (in base pairs) of products is shown on the left; product schematics are shown on the right. The %MSI_AS_ was calculated using ImageJ. Asterisks mark non-specific products. RS = Roifman syndrome; LWS = Lowry–Wood syndrome; EOCA = early-onset cerebellar ataxia. See also Supplementary Figures S9–S11.

Next, we wanted to evaluate whether inhibition of U4atac snRNA in these patients resulted in elevated exon skipping, as we had observed in our cell culture experiment (Figure 1A–C). Overall, we detected 66, 80 and 75 AS events around minor introns in healthy controls, unaffected Roifman syndrome carriers and unaffected Lowry–Wood syndrome carriers, respectively (Figure 6A; Dataset S6). This number was elevated in the individuals with Roifman syndrome (100 AS events), but not in the individual with Lowry–Wood syndrome (78 AS events) (Figure 6A; Dataset S6). In total, we detected 178 AS events around minor introns in one or more samples, of which 77 were elevated at least 2-fold in Roifman syndrome and/or Lowry–Wood syndrome compared to their unaffected carriers and healthy controls. Almost 50% of these upregulated AS events included a form of exon skipping, whereas 37% included the usage of a cryptic SS (Supplementary Figure S9A). In all, the level of AS (%MSI_AS_) was increased at least 2-fold for 39 AS events in both Roifman syndrome and Lowry–Wood syndrome (Figure 6B). Moreover, 8 AS events were exclusively elevated ≥2-fold in Lowry–Wood syndrome, whereas 33 AS events were uniquely elevated in Roifman syndrome (Figure 6B). In total, we identified 29 high-confidence AS events that were significantly upregulated ≥2-fold in Roifman syndrome compared to healthy controls and unaffected carriers (Supplementary Figure S9B). Several of these AS events were observed in the same MIGs, such as the skipping of the upstream 5′ exon (CAT3) combined with a cryptic 3′SS (CAT7) (Supplementary Figure S9C; Dataset S6). The combined usage of these AS events was validated by RT-PCR and Sanger sequencing for several MIGs (Figure 6C; Supplementary Figure S10). This also confirmed the RNA-seq findings that AS around minor introns generally occurs at higher levels in Roifman syndrome than Lowry–Wood syndrome (Figure 6C). Regardless, PCA on the %MSI_AS_ levels for all 178 detected AS events did separate all the individuals with Roifman syndrome and Lowry–Wood syndrome from the controls (Supplementary Figure S9D). Thus, even though the specific mutations in the U4atac snRNA may have a differential effect on the amount of AS around minor introns, they can all result in the production of aberrant MIG transcripts. This notion was further corroborated when we compared the specific AS events observed in the individuals with Roifman syndrome with those detected using a morpholino against U4atac snRNA. Specifically, we found that only 28% of the significantly elevated AS events in Roifman syndrome were also significantly elevated upon inhibition of U4atac snRNA with morpholinos (Supplementary Figure S2D). Hierarchical clustering of the samples based on AS levels then revealed that the control MO and U4atac MO samples were more similar than the U4atac MO samples and samples from individuals with mutations in U4atac snRNA (Supplementary Figure S11). Thus, this suggests that the primary driver of the clustering was the difference in cell type between the samples, which is in line with our previous finding that AS around minor introns is tissue specific in healthy humans (14).

Finally, we wanted to confirm whether mutations in components of the U11/U12 di-snRNP would also result in increased exon skipping in patients. An autosomal recessive n.84C>T mutation in the U12 snRNA has been linked to early-onset cerebellar ataxia and results in elevated minor intron retention (24). However, the effect of this mutation on AS around minor introns had not been studied (Supplementary Figure S9E) (24). Therefore, for those minor introns that were alternatively spliced in the *RNU4ATAC* RNA-seq data, we performed RT-PCR analysis on PBMCs from individuals with early-onset cerebellar ataxia, as well as their heterozygous parents and unrelated healthy controls (Figure 6C and D). We found that all of the AS events detected in the individual with Roifman syndrome were also identified in individuals with early-onset cerebellar ataxia (Figure 6C and D). In addition, a novel isoform for *TCP1* was detected in individuals with early-onset cerebellar ataxia from one family (Figure 6D; 270 bp). Comparison of the %MSI_AS_ values for the events around the minor introns of *TCP1*, *NCBP2*, *PFDN5* and *VPS35* revealed that AS was generally higher in individuals with early-onset cerebellar ataxia than the individuals with Roifman syndrome and Lowry–Wood syndrome (Figure 6C and D). Moreover, the minor intron retention events in *TCP1* and *VPS35* that we could detect in PBMCs from individuals with Roifman syndrome and Lowry–Wood syndrome were absent in individuals with early-onset cerebellar ataxia (Figure 6C and D). In all, these findings suggest that aberrant alternatively spliced MIG transcripts are part of the molecular pathogenesis underlying minor spliceosome-related diseases.

## DISCUSSION

### Exon-bridging interactions between the major and minor spliceosomes

The exon-definition model describes how long introns in vertebrates are recognized and spliced by the spliceosome (8,9). Specifically, it suggests that U1 and U2 snRNPs do not recognize the 5′SS and BPS of the same intron, but instead form a molecular bridge across the exon interrupting two introns (65). This exon-definition complex then needs to be remodeled into an intron-spanning complex to splice the intron. While it was known that the U4/U6.U5 tri-snRNP could be recruited to an exon-definition complex, it was only shown recently how this recruitment can then result in the formation of an intron-spanning spliceosomal complex (66,67). Even though this model clearly explains how introns can be spliced in genes exclusively dependent on the major spliceosome, genes that contain a minor intron pose a problem. Given that most MIGs are predominately made up of major introns and only contain one or two minor introns, splicing of these introns according to the exon-definition model requires cross-talk between the major and minor spliceosomes (14). Evidence that minor introns were also spliced according to the exon-definition model was first provided in 1996, when Wu and Krainer discovered that binding of U1 snRNP to a downstream major-type 5′SS would enhance the splicing of an upstream minor intron in the *SCN4A* splicing construct (15). These findings were later also confirmed in plants (9,15,68). However, it remained unclear whether binding of U11/U12 di-snRNP to minor introns would act analogous to U1 snRNP, and enhance the splicing of the upstream major intron. We found that loss of U11 snRNA did indeed result in increased exon skipping, suggesting that lack of U11 binding to the 5′SS of minor introns resulted in failure to use the 3′SS of upstream major introns by the major spliceosome (Figure 1D–F). In other words, these data suggest that U11 snRNP plays a role in mediating exon-definition interactions for a subset of MIGs. Similarly, binding of U11 snRNP to U11 snRNP splicing enhancer sequences in introns of *SNRNP48* and *RNPC3* has been shown to activate the usage of an upstream major-type 3′SS (17). Thus, the upregulation of exon skipping around minor introns in response to U11 loss can easily be reconciled by invoking an analogous function of U11 snRNP to U1 snRNP in maintaining exon-definition interactions (Figure 1D–F). To our surprise, AS around minor introns was also elevated in response to inhibition of U12, U4atac and U6atac snRNAs (Figure 1A–C). Thus, we propose that besides the U11/U12 di-snRNP, the successful assembly and activity of the entire minor spliceosome might regulate the major spliceosome.

### U11-59K interacts with the major spliceosome

**Figure 7.**
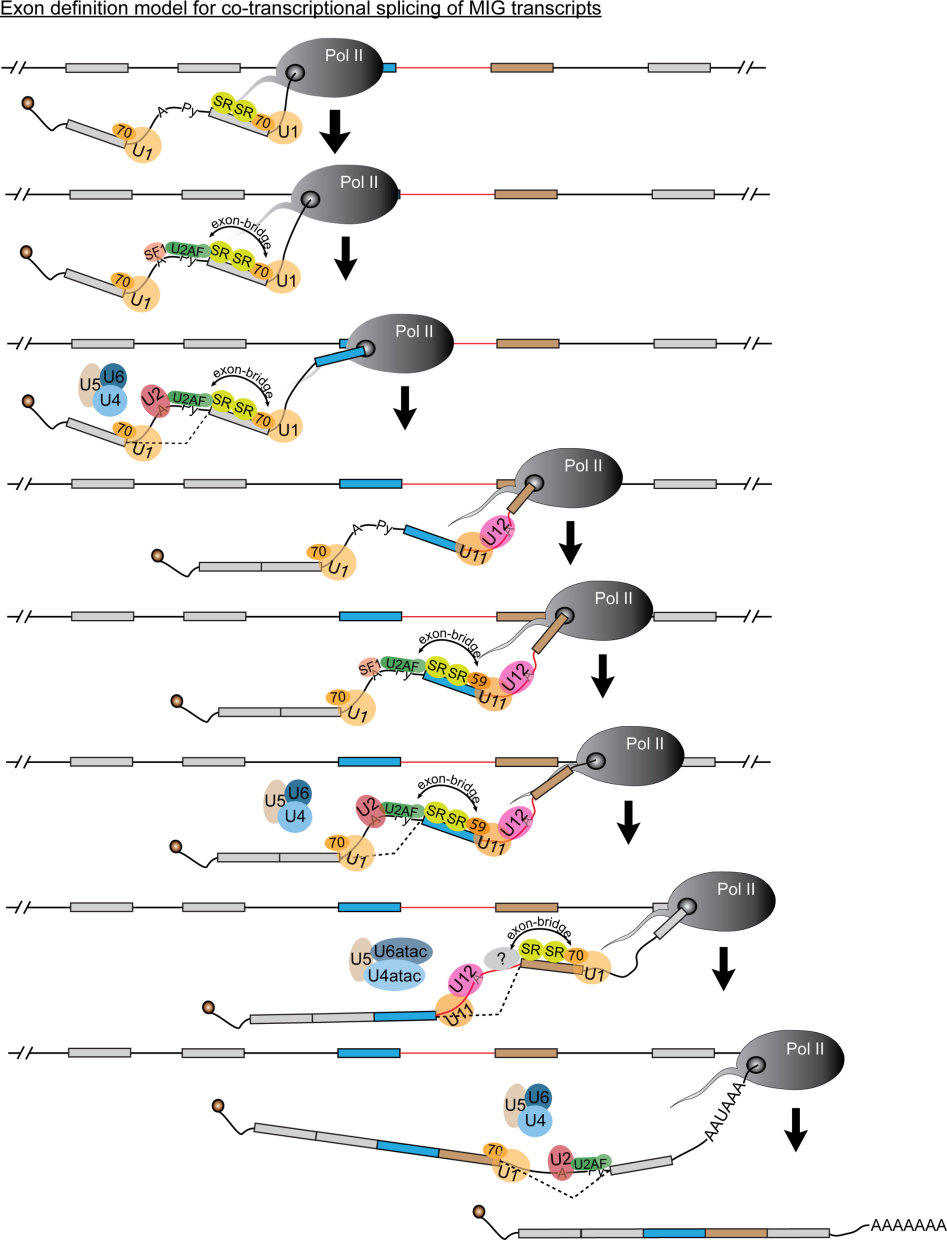
Exon-definition model for splicing of introns in MIGs. Schematic depicting the co-transcriptional splicing of major and minor introns in MIG transcripts. Minor intron is shown as red line; major introns are shown as black lines.

The exon-bridging interactions between major spliceosomes relies on U1-70K, which suggests that similar splicing and/or AS factors might play a role to establish exon-definition interactions between the minor and major spliceosomes (44). Based on previous reports that *SNRNP35* (U11-35K) might be the functional analog of U1-70K, we expected that its downregulation by siRNA would result in elevated AS of the *Mlst8* minigene construct (16,69). Indeed, exon skipping was upregulated >2-fold upon knockdown of snRNP35, but we saw the largest increase in exon skipping upon downregulation of *PDCD7* (U11-59K), *RNPC3* (U11/U12-65K) and *ZRSR2* (Urp) (Figure 3A). While this difference might also be attributable to variability in knockdown efficiency, and therefore does not exclude a potential role of U11-35K in exon definition, we here focused on the genes with the biggest effect. ZRSR2 is a component of both the major and minor spliceosomes, and is thought to play a role in 3′SS selection of minor introns (45,70). In contrast, both PDCD7 and RNPC3 are part of the seven unique minor spliceosome proteins (69). Since PDCD7 is part of the U11 snRNP and has a highly specific antibody, which gave us confidence in the efficacy of the siRNA, we focused on this protein (Supplementary Figure S5B). Immunoprecipitation of PDCD7 followed by LC–MS/MS resulted in the detection of many interacting proteins that included both RNA splicing factors and non-splicing factors (Figure 3E–H; Supplementary Figure S6). This finding suggests that PDCD7 plays additional roles besides minor intron splicing, which is also evidenced by findings that PDCD7 transactivates E-cadherin expression and is involved in apoptosis of T cells (71,72). Our focus here was the role of PDCD7 as part of the minor spliceosome, and therefore we curated those interacting proteins known to play a role in RNA splicing. This revealed that PDCD7 interacts with proteins of the SR-protein family, but also SF1, SF3B1, U2AF1 and U2AF2 (Figures 3E–H and 7). These proteins have all been shown to be crucial components of the exon-bridging complex formed between major introns (65). Our data are therefore compatible with a model in which PDCD7 acts as a molecular bridge between the minor and major spliceosomes, to maintain exon-definition interactions in MIGs (Figure 7). Together, these findings for the first time reveal a potential reciprocal regulation of the two spliceosomes to regulate MIG expression.

### Alternatively spliced MIG isoforms are not degraded and bound to polysomes

While we and others have begun to elucidate how introns in MIGs are spliced, and have increased our understanding of the consequences of disrupting the exon-definition interactions between major and minor spliceosomes, it remained unclear how the expression of the resulting aberrant isoforms would impact disease pathogenesis. Bioinformatics analysis showed that many of the AS events observed in the U11 cKO resulted in a premature stop codon, which was confirmed by Sanger sequencing (Figure 1D and E; Supplementary Figures S3B and S7A). Premature stop codons located ≥50 nt upstream of the last exon–exon junction are thought to be degraded by the NMD pathway, which was predicted for approximately half of all aberrant MIG transcripts (37). Unexpectedly, these alternatively spliced MIG transcripts escaped nuclear degradation, and were instead bound to polysomes (Figure 4A–C). Initially, we hypothesized that their presence without concomitant downregulation at the gene level could be explained by the fact that several crucial components of the NMD pathway are MIGs, such as *Upf1*, *Ncbp1*, *Ncbp2* and *Ice1* (18,73,74). As such, inhibition of the minor spliceosome could also affect the splicing of minor introns in these genes and affect their function (Figures 1C and 6C and D) (18). However, the alternatively spliced transcripts of *hnRNPh1* and *Srsf3*, which are known NMD targets, were not upregulated in the U11 cKO, suggesting that the NMD pathway is not affected by minor spliceosome inhibition (Figure 4D) (52). Instead, we found that inhibition of the NMD pathway *in vivo*, through addition of cycloheximide, barely affected the level of alternatively spliced MIG transcripts in the U11 cKO (Figure 4D). This suggests that these aberrant MIG transcripts, despite containing a premature stop codon, are not degraded by the NMD pathway. It remains to be known whether these MIG transcripts possess a feature that allows them to escape NMD, or whether the ‘50–55 nt rule’ is not as stringent as previously thought. The fact that alternatively spliced MIG transcripts were not subject to NMD, while detected in polysomes, suggested that they were translated (Figure 4C and D). However, since the resulting protein products are generally small and lack crucial protein domains, it is possible that they are quickly degraded by protein control mechanisms (Supplementary Figure S7A).

### Relationship between AS around minor introns and severity of symptoms in minor spliceosome-related diseases

While only responsible for the splicing of 0.5% of all introns, the importance of the minor spliceosome in development is underscored by the diseases MOPD1, Roifman syndrome, Lowry–Wood syndrome, early-onset cerebellar ataxia and IGHD (24,30,58–61). The underlying molecular etiology in all these diseases is inhibition of the minor spliceosome, even though the disease-causing mutations are found in different minor spliceosome components. Consequently, these diseases can be characterized by several overlapping symptoms, such as microcephaly, developmental delays and growth retardation (57). In the *RNU4ATAC*-related diseases, these symptoms are found on a spectrum of severity, where individuals with MOPD1 are most severely affected and individuals with Lowry–Wood syndrome the least (22). This suggests the presence of genotype–phenotype relationships that might be informed by the effect of the specific mutations on the secondary structure of U4atac snRNA and therefore inhibition of the minor spliceosome. As a result, differences in the level and types of minor intron mis-splicing might contribute to the differences in phenotype severity. Previous transcriptomic analyses of individuals with Roifman syndrome and MOPD1 have revealed widespread minor intron retention in a shared subset of MIGs, but the authors of these studies also noted that these samples were hard to compare due to differences in age, cell type, sequencing depth and sex (63). Fortuitously, the transcriptomes we sequenced from individuals with Roifman syndrome and Lowry–Wood syndrome were all from PBMCs of males, and contained a similar sequencing depth. However, it must be noted that the cDNA library preparation differed between the individuals with Roifman syndrome. Regardless, our data allowed us to compare the effect of the different *RNU4ATAC* mutations on the retention and AS of minor introns. Specifically, the individual with Roifman syndrome first described in this manuscript contains one variant in the stem II loop of U4atac, which is important for base pairing with U6atac snRNA, while the other variant is located in the Sm binding domain of U4atac snRNA, which is required for the loading of the Sm ring (Supplementary Figure S8A). The two individuals with Roifman syndrome that had previously been described both contained one variant in the stem II loop, as well as one variant in the 5′ stem loop, which binds to the tri-snRNP proteins 15.5K and PRPF31 (Supplementary Figure S8A) (30,75,76). In contrast, both mutations in the individual with Lowry–Wood syndrome were located in or near the Sm binding domain of U4atac (Supplementary Figure S8A). Our analysis revealed a significant overlap in the number of MIGs that showed minor intron retention in both Roifman syndrome and Lowry–Wood syndrome (Figure 5C). However, the number of retained minor introns was higher in the clinically more severe Roifman syndrome compared to Lowry–Wood syndrome (Figure 5C). Previous transcriptomic analysis had already shown that the number of retained minor introns was elevated in MOPD1 compared to Roifman syndrome (63). Thus, these findings support the notion of genotype–phenotype relationships that are informed by the level of minor intron mis-splicing. Importantly, our analysis included unaffected heterozygous carriers for Roifman syndrome and Lowry–Wood syndrome, which showed that one mutant *RNU4ATAC* allele is not sufficient to result in aberrant minor intron splicing (Figure 5A and B).

In addition to minor intron retention, we observed a large number of AS events in the individual with Roifman syndrome, but not in the individual with Lowry–Wood syndrome (Figure 6A). This suggests that the manner in which U4atac snRNA, and in turn the minor spliceosome, is disrupted might inform whether minor introns are retained and/or alternatively spliced. Specifically, our results suggest that disruption of the Sm binding domain in U4atac, which might reduce the levels of mature U4atac snRNP but not affect the base pairing with U6atac snRNA, would inhibit the minor spliceosome such that it results in minor intron retention (77). In contrast, mutations in stem II loop of U4atac snRNA, which is important for base pairing with U6atac snRNA, inhibit the minor spliceosome such that AS around minor introns was elevated (77). Thus, the maintenance of base-pairing interactions between U4atac and U6atac snRNAs might be important for maintenance of exon-definition interactions. Finally, the elevated expression of alternatively spliced MIG transcripts in individuals with early-onset cerebellar ataxia underscored the importance of U12 snRNA in exon-definition interactions (Figure 6D).

Overall, our work is revealing the complex regulation of splicing and AS of minor introns through coordinated action of the major and minor spliceosomes, which is in line with the exon-definition model. Given that minor introns and the minor spliceosome evolved very soon after major introns, and are highly conserved across species, the regulated cross-talk is consistent with the idea of co-evolution of the exon-definition interactions (78). Inherent to these interactions is the means to regulate the splicing and AS of many MIGs that are essential for survival, cell cycle and other functions (79). As the involvement of the minor spliceosome in diseases such as amyotrophic lateral sclerosis, myelodysplastic syndrome and others is being discovered, understanding the regulation of MIG expression will prove to be invaluable (80–82).

## DATA AVAILABILITY

The datasets generated and analyzed during this study are available in the following databases:

- RNA-seq data: Gene Expression Omnibus GSE96616.
- RNA-seq data: Gene Expression Omnibus GSE151142.
- Mass spectrometry data: ProteomeXchange Consortium via the PRIDE partner repository with dataset identifier PXD019428.

## Supplementary Material

gkab118_Supplemental_Files
