# Supplementary material for: Disruption of exon-bridging interactions between the minor and major spliceosomes results in alternative splicing around minor introns
Source: Nucleic Acids Res. 2021 Feb 28;49(6):3524–45. doi: 10.1093/nar/gkab118 (PMC8034651; doi:10.1093/nar/gkab118)
Supplement: gkab118_Supplemental_Files [file gkab118_supplemental_files.zip › Supplemental Information NAR resubmission round 2 v3.docx]

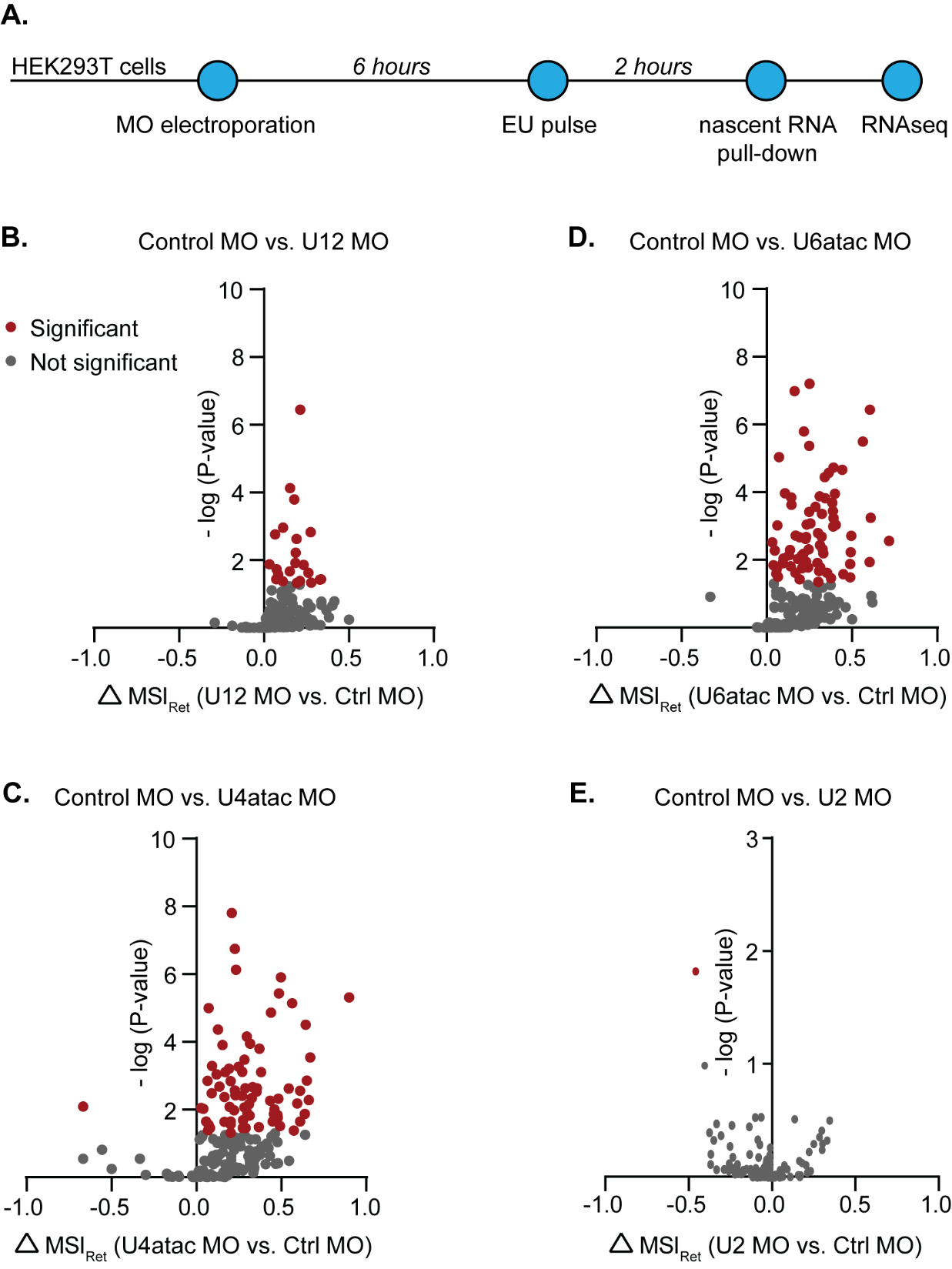


**Figure S1. Inhibition of minor spliceosome snRNAs through morpholinos results in elevated minor intron retention. (A)** Schematic of experimental design for morpholino (MO) electroporations. **(B-E)** Volcano plot showing the delta MSI_Ret_ for retained minor introns upon inhibition of U12 snRNA **(B)**, U4atac snRNA **(C)**, U6atac snRNA **(D)** and U2 snRNA **(E)**. Significance was determined by one-way ANOVA, followed by post-hoc Tukey test.


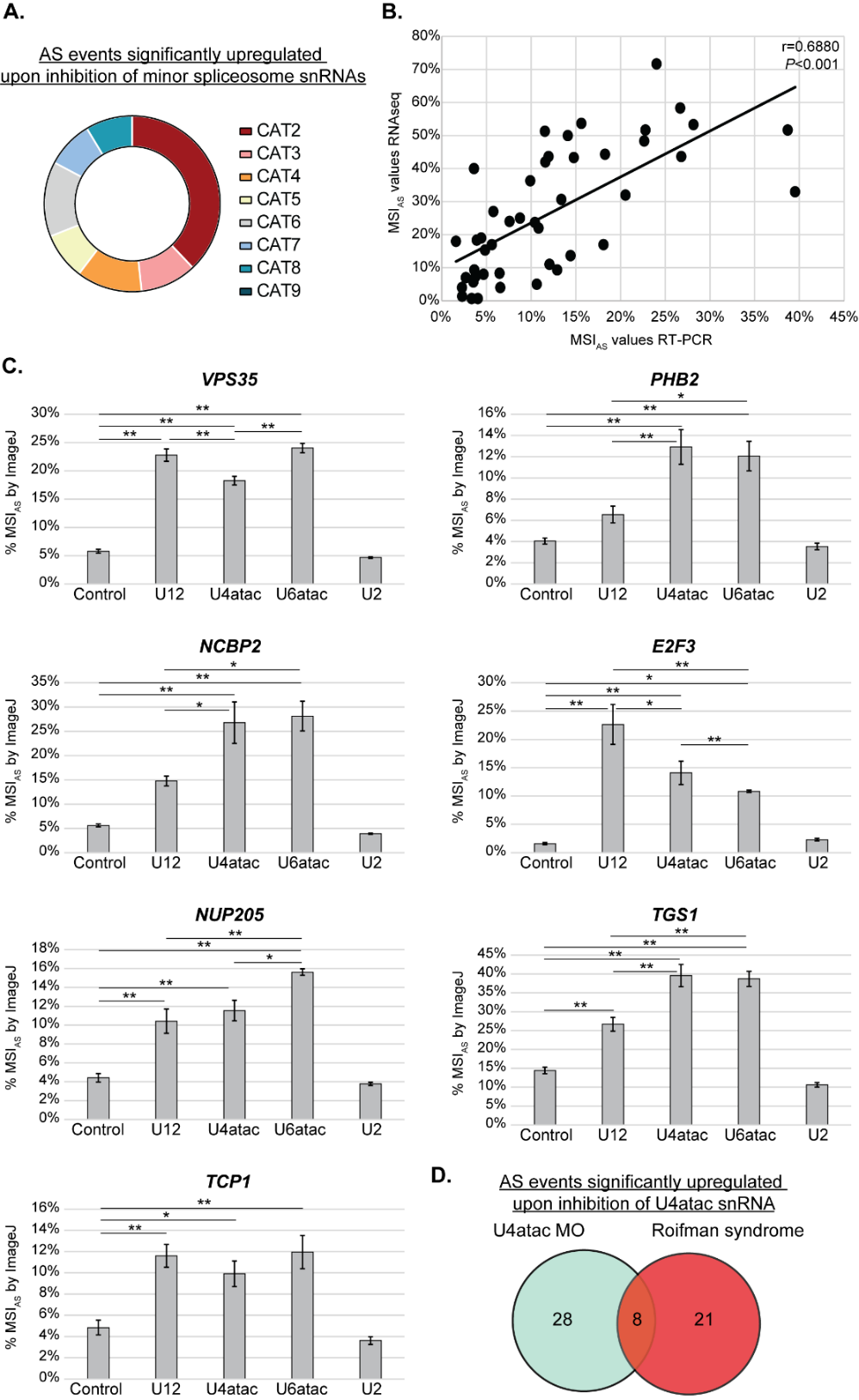


**Figure S2. Inhibition of minor spliceosome snRNAs through morpholinos results in elevated alternative splicing. (A)** Doughnut chart with the distribution of different AS events that are significantly upregulated upon minor spliceosome inhibition. **(B)** Scatterplot with correlation between %MSI_AS_ values determined by RNAseq and ImageJ quantification of RT-PCR products. **(C)** Bargraphs with %MSI_AS_ values of RT-PCR analysis, as calculated using ImageJ. Significance was determined using one-way ANOVA, followed by post-hoc Tukey test. **(D)** Venn diagram with number of AS events significantly elevated upon U4atac MO and in individuals with Roifman syndrome. MO=morpholino. ***=***P*<0.05; ****=***P*<0.01


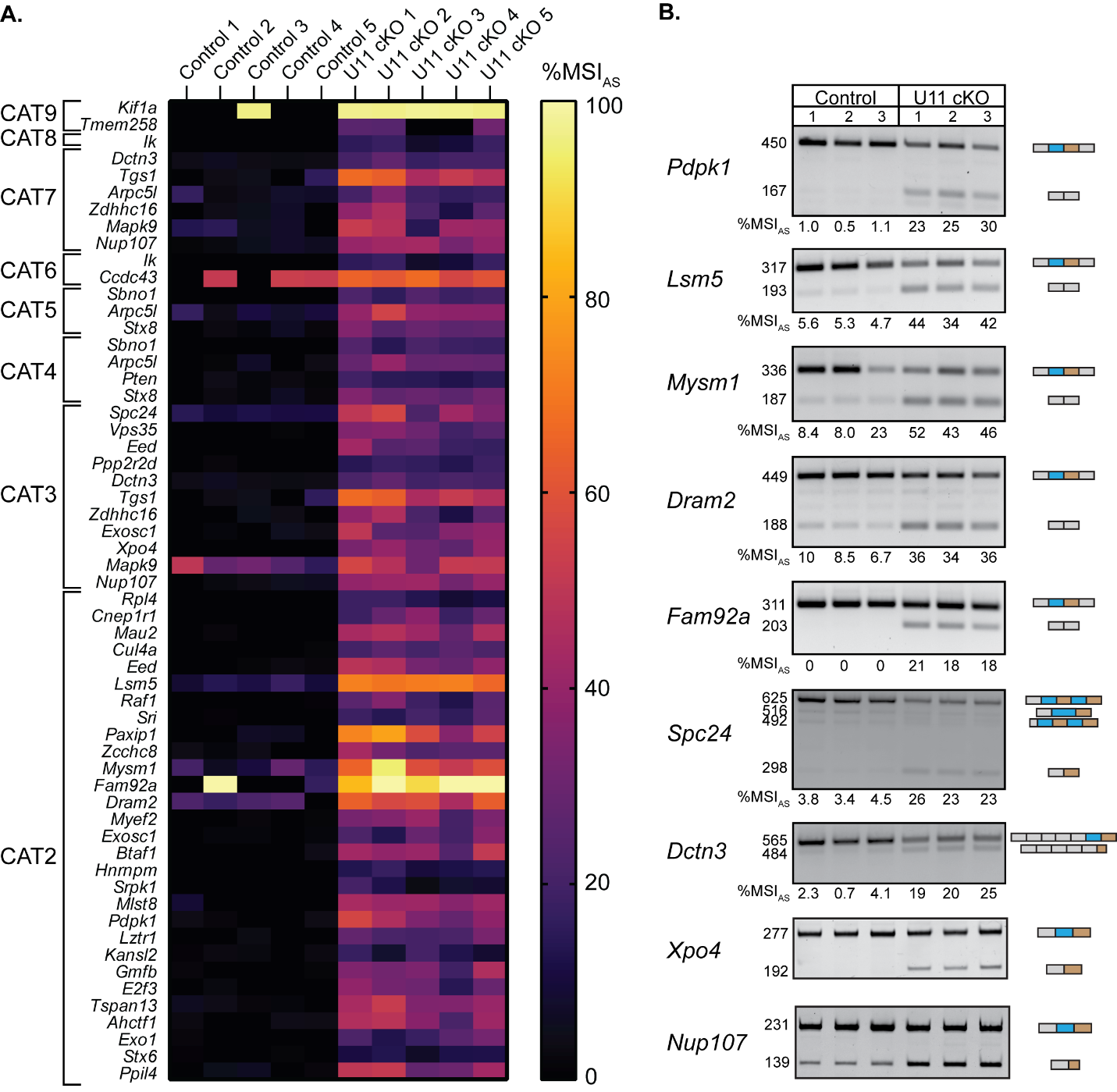
**Figure S3. Loss of U11 results in elevated exon skipping. (A)** Heatmap of MSI_AS_ values for AS events significantly upregulated in the U11 cKO. **(B)** Gel images of RT-PCR products resulting from AS around minor introns. Product size is shown on the left; product schematics are shown on the right. The %MSI_AS_ was calculated using ImageJ.

**
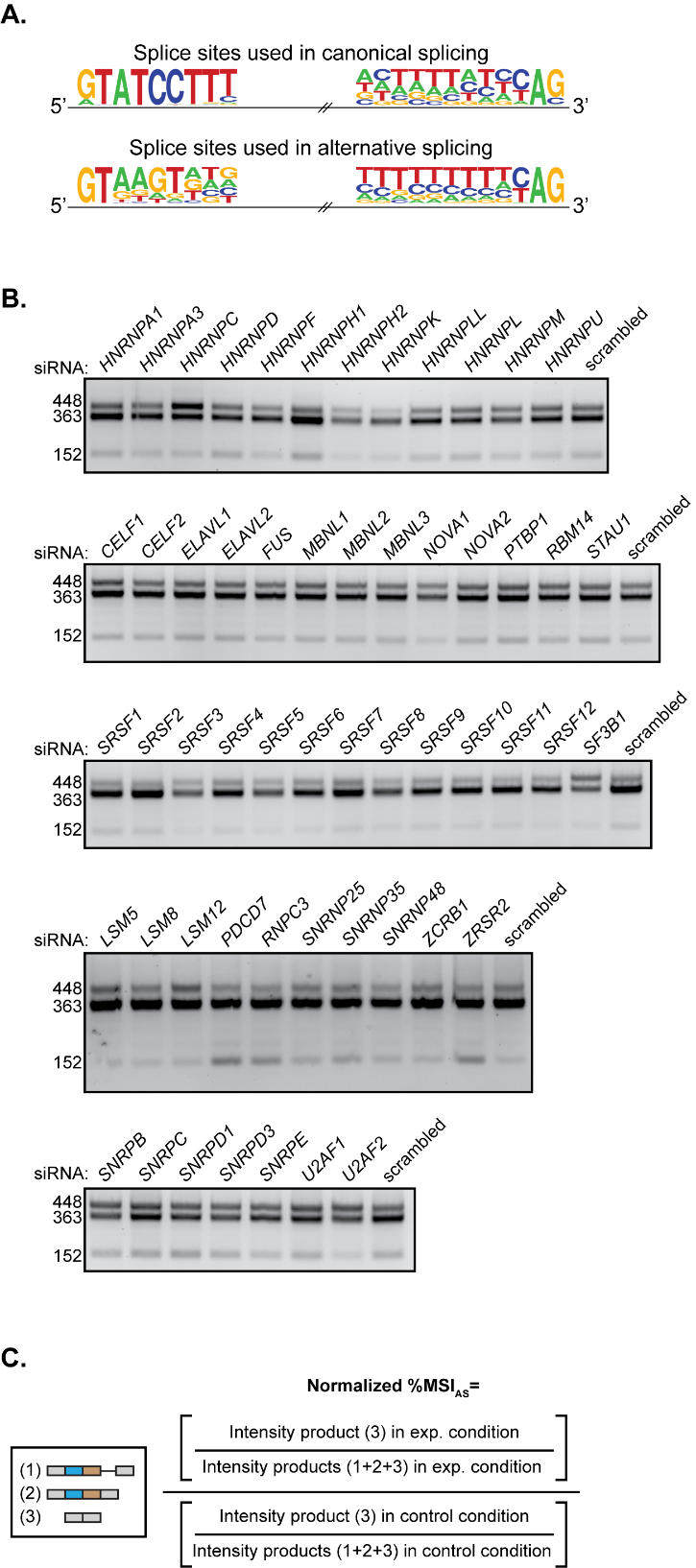
**

**Figure S4. Alternative splicing around minor introns is executed by the major spliceosome and regulated by minor spliceosome proteins. (A)** Frequency logos of the 5’ SS and 3’ SS of alternatively spliced minor introns in the U11 cKO. The annotated consensus sequences of all minor introns with significantly elevated AS in the U11 cKO were used to construct the top plot. The bottom plot was generated by extracting the splice site sequences that were used in case of alternative splicing. **(B)** Agarose gel images of the *Mlst8* splicing reporter RT-PCR products resulting from downregulation of proteins in the siRNA screen. **(C)** Equation used to determine the normalized %MSI_AS_ in the siRNA screen.


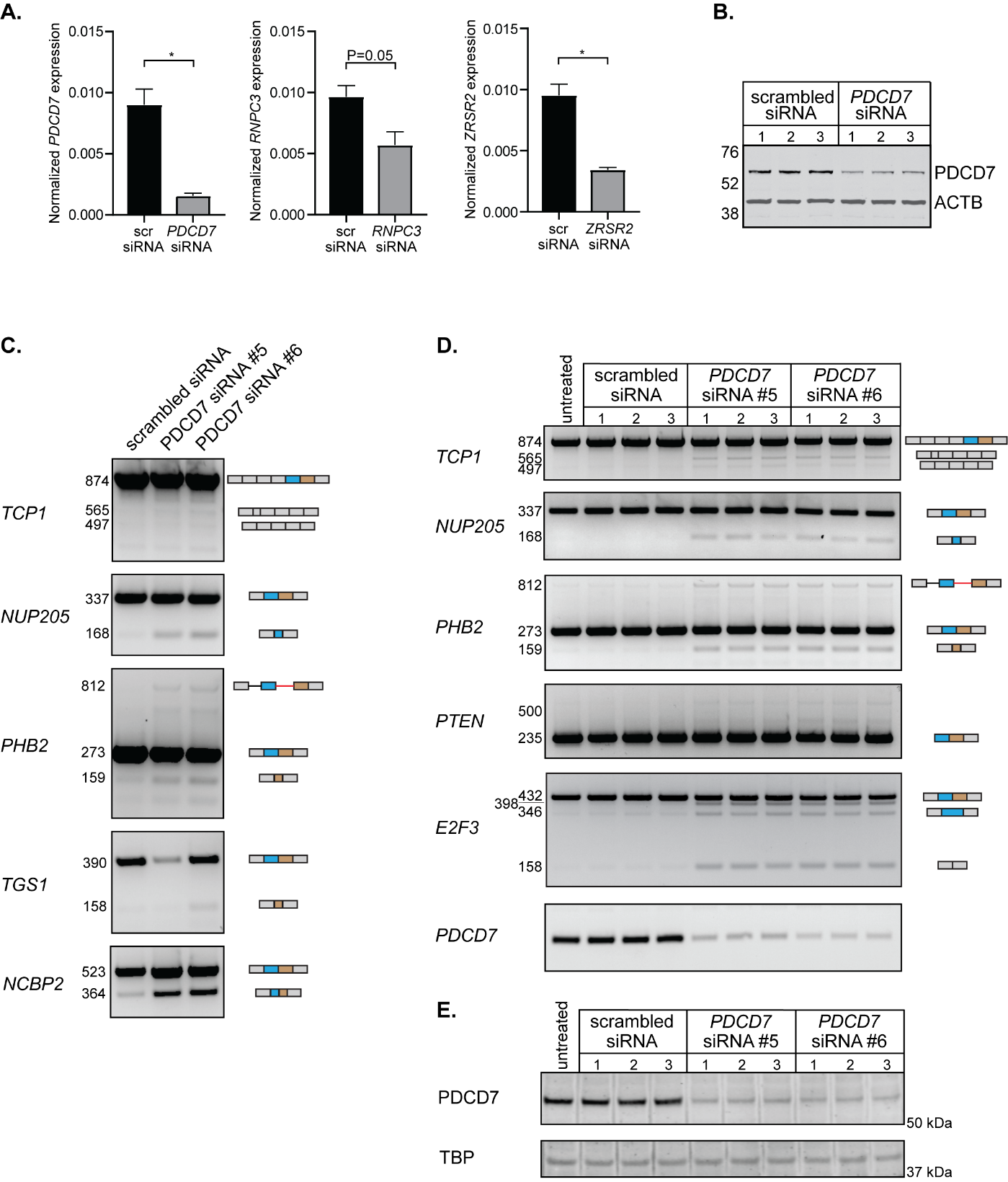


**Figure S5. Knockdown of PDCD7 results in higher levels of AS around minor introns in lung adenocarcinoma cells than HEK293. (A)** qRT-PCR results with expression levels of *PDCD7, RNPC3* and *ZRSR2,* normalized to *GAPDH*. Significance was determined by Student’s t-test. **(B)** Immunoblot for PDCD7 and Actin after siRNA transfection in HEK293 cells. **(C-D)** Agarose gel images of RT-PCR products resulting from AS around minor introns after knockdown of PDCD7 in HEK293 **(C)** and A549 **(D)** cells. Product size is shown on the left; product schematics are shown on the right. **(E)** Immunoblot for PDCD7 and TBP after siRNA transfection in A549 cells.

**
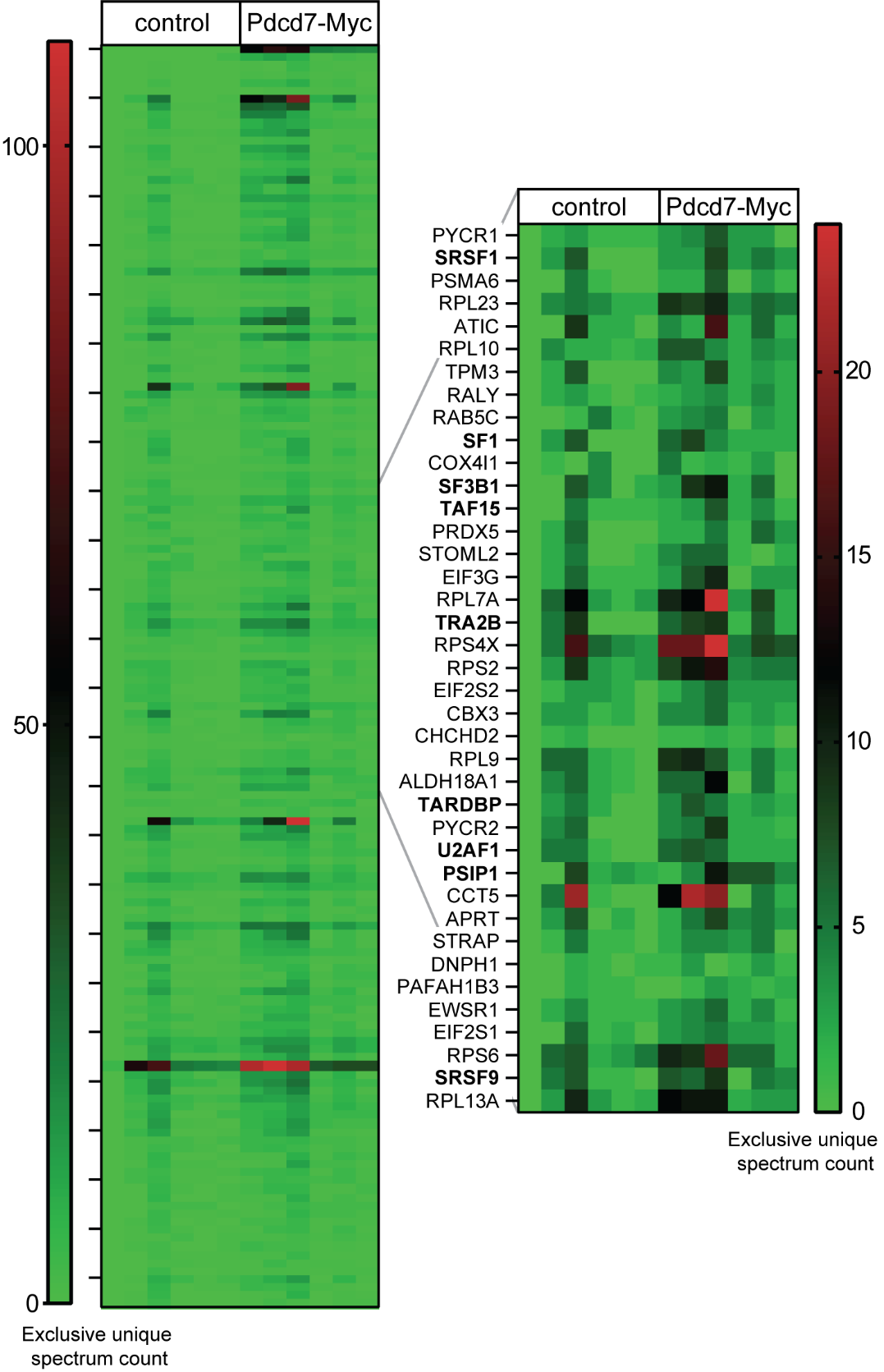
**

**Figure S6.** **Identification of proteins interacting with U11-59K.** Heatmap of exclusive unique spectrum counts of all proteins >2-fold enriched in the Pdcd7-Myc IP samples. Zoomed in subset shown on the right. Proteins involved in splicing are shown in bold.


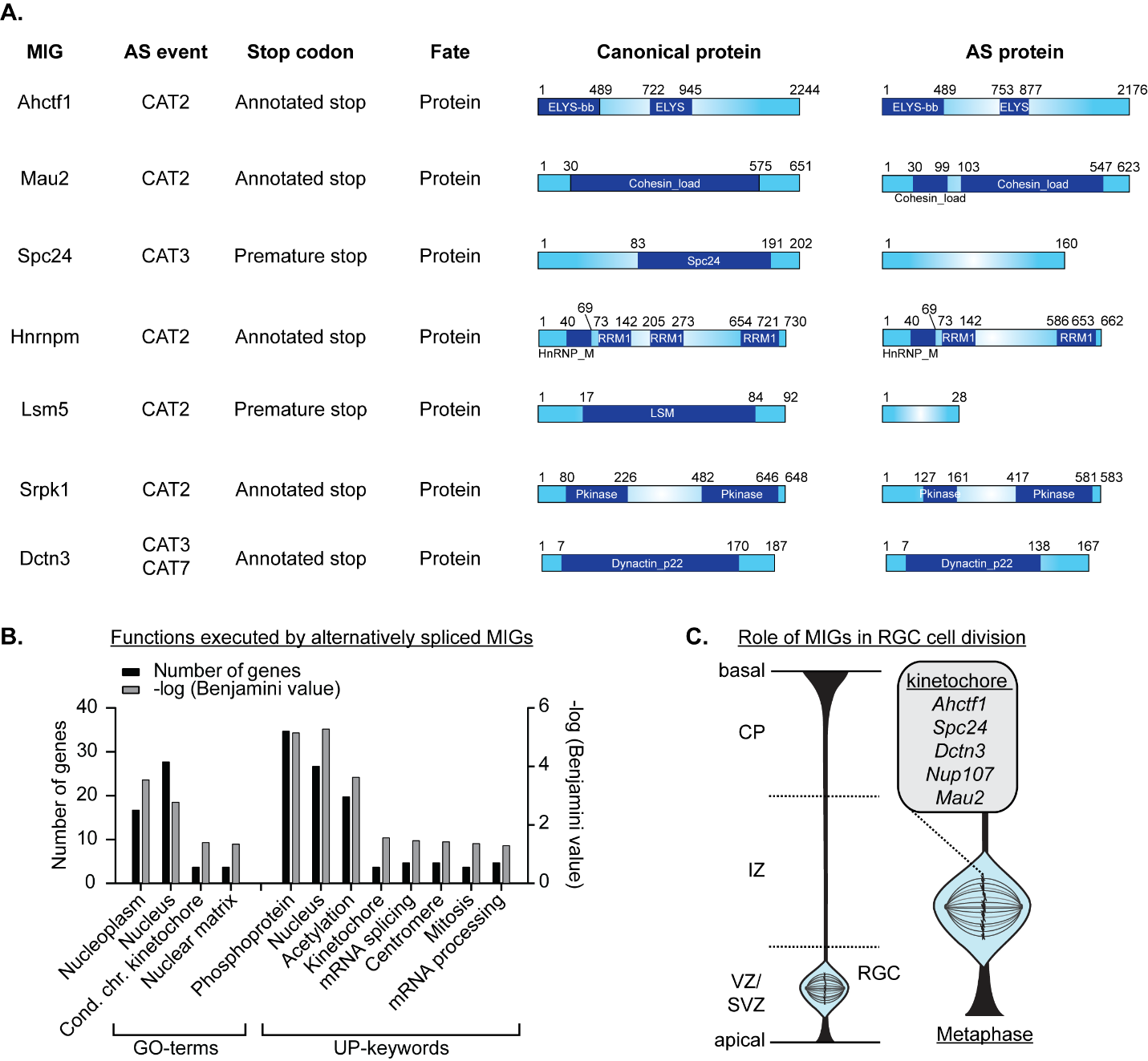
**Figure S7. Alternatively spliced MIG isoforms play a role in mitosis. (A)** Schematics showing altered protein domains due to AS around minor introns. Numbers above indicate amino acids that encode for each protein domain. **(B)** Bargraph with functional annotation of MIGs with significantly elevated AS around minor introns in the U11 cKO. **(C)** Schematic of layers in developing cortex and the location of radial glial cells (RGCs). On the right is a schematic of an RGC in metaphase and the MIGs enriching for the kinetochore function in **(B)**. CP=cortical plate; IZ=intermediate zone; VZ=ventricular zone; SVZ=subventricular zone.


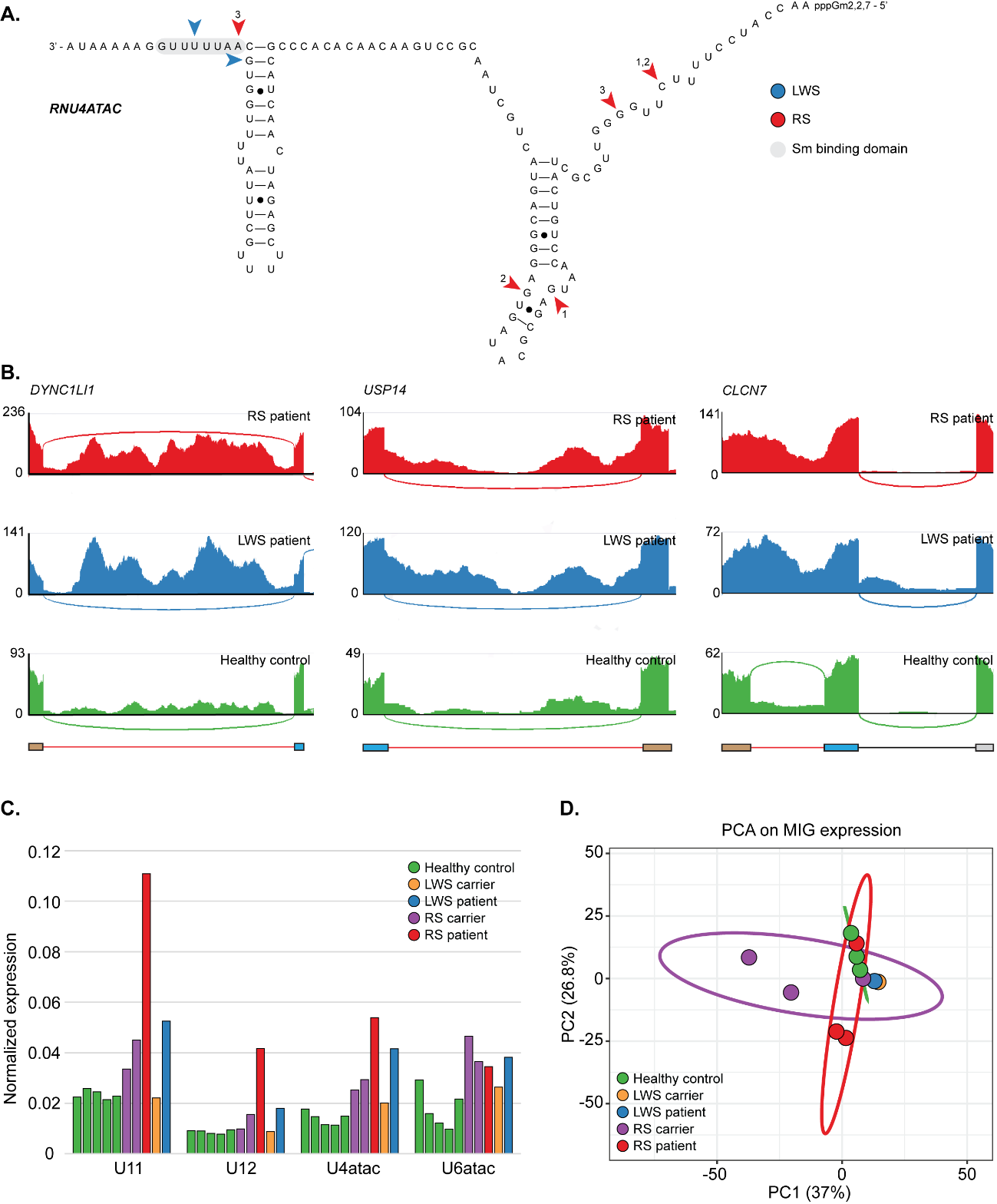


**Figure S8. The effect of *RNU4ATAC* mutations on minor intron retention, minor spliceosome snRNA levels and MIG expression. (A)** Schematic of the secondary structure of U4atac snRNA and the locations of the mutations in the RS and LWS patient. **(B)** Sashimi plots showing read coverage across minor introns. **(C)** Bargraphs with normalized expression of minor spliceosome snRNAs. **(D)** Principal component analysis (PCA) on TPM values of MIGs in patients and controls. Prediction ellipse is drawn such that the probability is 95% that a new observation from the same condition falls inside the ellipse. RS=Roifman syndrome; LWS=Lowry-Wood syndrome.


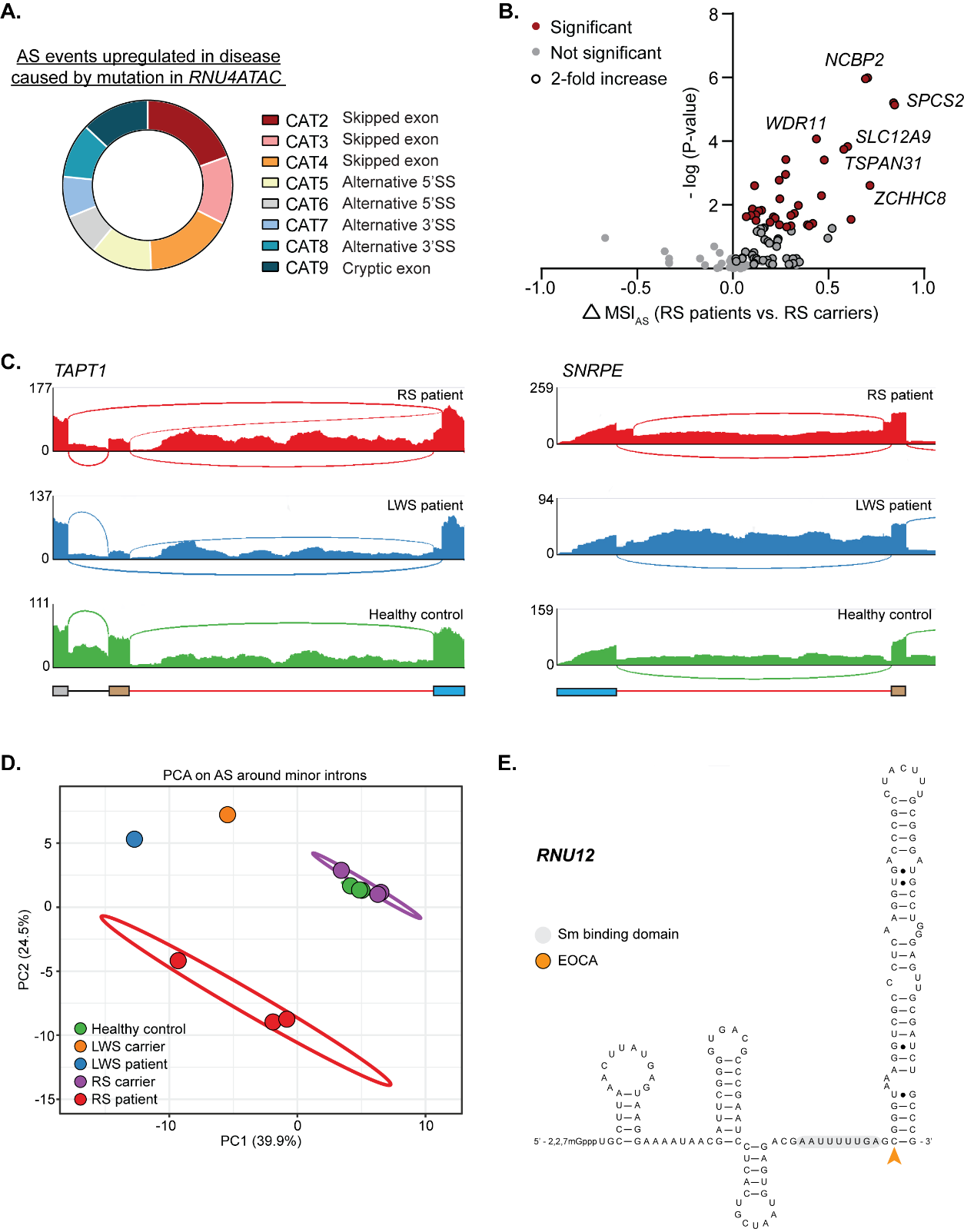


**Figure S9. Alternative splicing around minor introns is upregulated in Roifman Syndrome. (A)** Doughnut chart with the distribution of different AS events that are upregulated in the RS and/or LWS patient. **(B)** Volcano plot of alternatively spliced MIGs in RS. **(C)** Sashimi plots showing combined AS usage around minor introns. **(D)** Principal component analysis (PCA) on %MSI_AS_ in patients and controls. Prediction ellipse is drawn such that the probability is 95% that a new observation from the same condition falls inside the ellipse. **(E)** Schematic of the secondary structure of U12 snRNA and the location of the mutations in the early-onset cerebellar ataxia (EOCA) patients. RS=Roifman syndrome; LWS=Lowry-Wood syndrome.


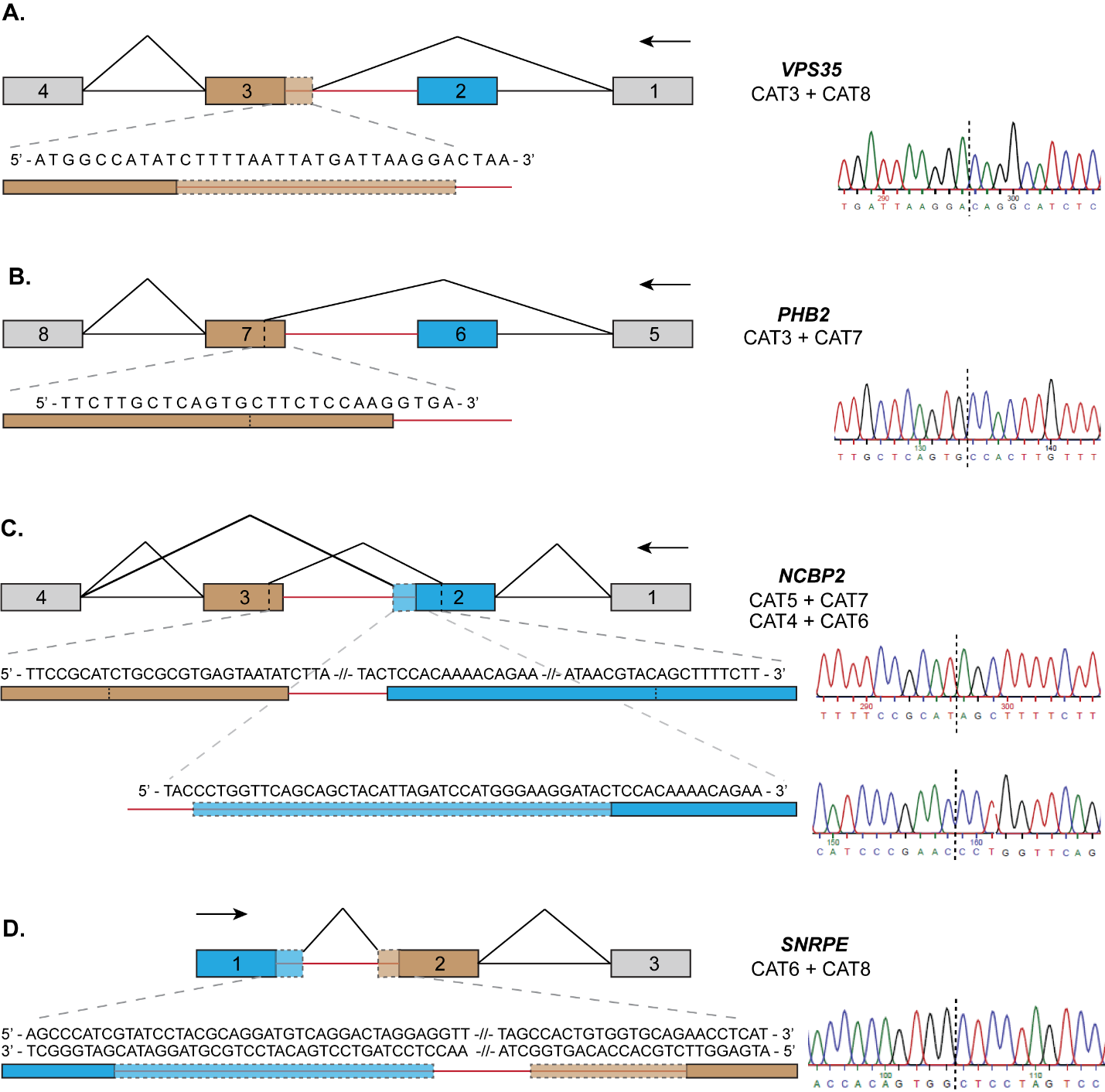


**Figure S10.** **Sanger sequence confirmation of alternatively spliced MIG transcripts in Roifman syndrome and Lowry-Wood syndrome. (A-D)** Gene schematic and nucleotide sequence of alternatively spliced junctions with the supporting chromatograms for *VPS35* **(A)**, *PHB2* **(B),** *NCBP2* **(C)** and *SNRPE* **(D)**.


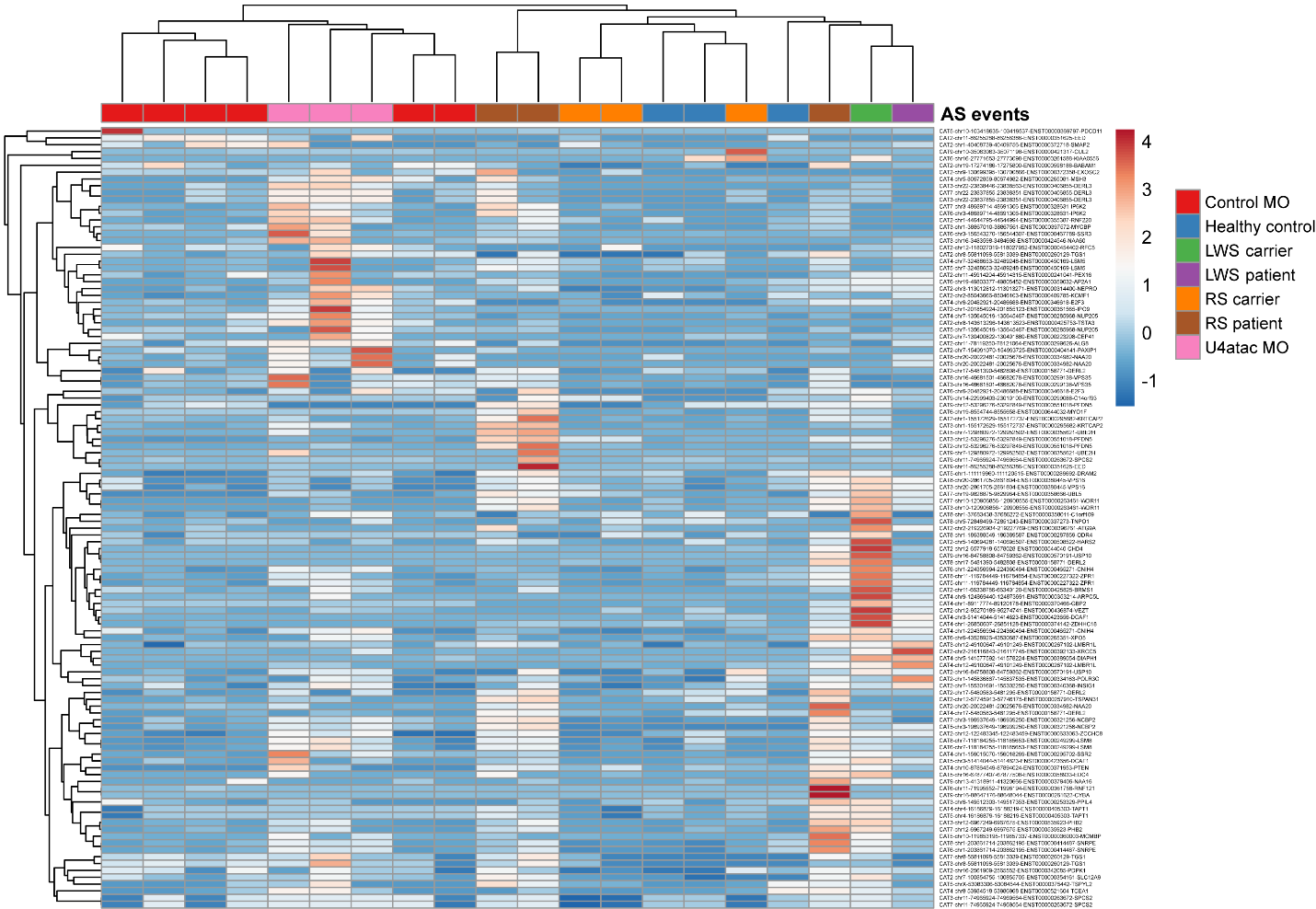


**Figure S11. Cell-type differences between patient and morpholino samples drive two main clusters.** Hierarchical clustering of samples with mutation in *RNU4ATAC* and samples electroporated with U4atac morpholino. Heatmap represents the levels of alternative splicing around minor introns in each sample.

**Table S1. Primer sequences.**

| **Gene** | **Forward primer** | **Reverse primer** |
| --- | --- | --- |
| *TCP1* | TATCGCTGCCGTCAAGATGGAGG | AATAACATTGGCACCAGTTGCCAGGAT |
| *NEPRO* | GCAGTCCTTTACAGCAATCACAACAGA | TTGAATCCTAGCGACCTCTTGAAGCAAT |
| *NCBP2* | ATGTCGGGTGGCCTCCTGAAG | AGCAAATTCAACAGGCCAAAGGAGTGTTT |
| *PFDN5* | GCTAGAAATGCTCAAGAACCAGCTGG | AGGGCTGTGAGCTGCTGAATCTTC |
| *PHB2* | ATCACAGAGCTGAGCTTTAGCCGAGA | TGTGAGATAGATACGATTCTGTGATGTGG |
| *VPS35* | GGCTGTGAAGGTCCAGTCATTCCAA | CACACCACGGCACATTTCTACCAAATC |
| *TGS1* | CTGTGGAGTTGGAGGAAATACCATTCAG | TGTTCTATTTCCACTTGCCCTCCAGG |
| *NUP205* | GAGCAGCTTCCCCCAGATGAGATA | TCACTATAGCCAGCCCACTTCTAAAATCT |
| *E2F3* | GGATTTGAACAAGGCAGCAGAAGTGC | ATGGGCCCTTGGGTACTTGCCAAAT |
| *PSMA1* | TTGCGGGGCTTACTGCTGATGCTA | GGACAGGTTTGGAAAATGTGAGGGC |
| *SNRPE* | GCGGGTGTGCTCTTTGTGAAATTCCA | CTCATAGAGCCACACCTGAATCCG |
| *PDCD7* | ACGCATCATCTTCAGCGACT | ACTGTCGGAAAGGCTCCAAG |
| *Nup107* | GCAGTCAAAGAGTTGCAAGGTTCTAGG | AAGGCTGTCGAAAGGAGCTTGGC |
| *Arpc5l* | AACACACTGTCCTCACGCTTCCG | AGTGACTGCACAGCCTGCTCAATTTC |
| *Fam92a* | CGAAATCAACCTGTATGCCTCTACCGA | TGCGGGTAGCATCAATCGTGGCT |
| *Spc24* | GATCAGTCATGGCGGCTTTCCG | TCAAGGCTCTGGCTCCCACGT |
| *Dctn3* | AACGATGGCGGCTCTGACCGAT | TCATTCCTCTGCTGGCTTCACTTGC |
| *Pdpk1* | GGGAAAATTCTTGGCGAGGGCTCTT | GCTCTCTGGGGATAACACTTTTGCTGT |
| *Lsm5* | TGTCCTTAGCCACTTCCGGCGT | GTCGAGGAAATCCATTCATACTTCAGGC |
| *Mysm1* | TGTGGATGTGGAAGGAGATGTTGCG | GTCCACTTTACTGAATGACTGGCTGGTT |
| *Dram2* | GCTGGCCTTGTACTTGGGATACTGA | CCATGTAAATTGGCTTCCACCCGC |
| *E2f3* | TGCTCAAGGTGCAGAAGAGGAGGATT | CTGGACACAAGTAAACCTCAATGGGC |
| *Mlst8* | CTAAGGCAGAGTGCAGAGCGTGTG | TATACATCCAGCGACCATCCTCGTGA |
| *Xist* | TTTGTGCTCCTGCCTCAAGAAGAAG | AATAGGTCGCCAGCACTGCAAA |
| *Vps35* | CCATCCAGGCTGTGAAGGTTCAGT | ATCAGTCAGGTAGACCTCCAAGTAGTG |
| *Xpo4* | TTAGAGGACATGAGCCAGGACAAGC | GCCTCGAGACAAAGCTGGCATACT |
| *RN7SK* | GCGATCTGGCTGCGACATCTGTCA | CCGAAGACCGGTCCTCCTCTATC |
| *RNU11* | CTTCTGTCGTGAGTGGCACACGTAG | CGGGACCAACGATCACCAGCT |
| *RNU12* | GGAAAATAACGATTCGGGGTGACG | CTCCCAGGCATCCCGCAAAGTA |
| *RNU4ATAC* | CATCCTTTTCTTGGGGTTGCGC | AAAGCAAAAGCTCTAGTTGATGCGGG |
| *RNU6ATAC* | GTTGTATGAAAGGAGAGAAGGTTAGCACTCC | AACGATGGTTAGATGCCACGAAGTAGG |

**Supplemental dataset 1. Minor intron retention upon inhibition of minor spliceosome snRNAs by morpholinos.**

**Supplemental dataset 2. AS around minor introns upon inhibition of minor spliceosome snRNAs by morpholinos.**

**Supplemental dataset 3. AS around minor introns in U11 cKO mice.**

**Supplemental dataset 4. Minor intron retention in Roifman and Lowry-Wood syndrome.**

**Supplemental dataset 5. MIG expression in Roifman and Lowry-Wood syndrome.**

**Supplemental dataset 6. AS around minor introns in Roifman and Lowry-Wood syndrome.**
